# Multi‐Target Modulation of Polyphenols in Diabetic Kidney Disease Therapeutics: A Comprehensive Review

**DOI:** 10.1002/fsn3.70882

**Published:** 2025-09-26

**Authors:** Esienanwan E. Efiong, Emmanuel Effa, Esther Peters, Ochuko L. Erukainure, Peter U. Amadi, Joshua Onyeka Ikebuiro, Sapna Sharma, Christoph Schmaderer, Kathrin Maedler, Emmy Tuenter, Harald Grallert

**Affiliations:** ^1^ Research Unit of Molecular Epidemiology Institute of Epidemiology, Helmholtz Zentrum München Neuherberg Germany; ^2^ Department of Pharmaceutical Sciences, Faculty of Pharmaceutical, Biomedical and Veterinary Sciences University of Antwerpen Antwerp Belgium; ^3^ Department of Biochemistry Faculty of Science, Federal University of Lafia Lafia Nigeria; ^4^ Division of Nephrology, Department of Internal Medicine, Faculty of Clinical Sciences University of Calabar Calabar Nigeria; ^5^ Faculty of Science, Masaryk University Brno Czech Republic; ^6^ Laser Research Centre, Faculty of Health Sciences University of Johannesburg Doornfontein South Africa; ^7^ Department of Pediatrics, Faculty of Medicine and Dentistry University of Alberta Edmonton AB Canada; ^8^ Department of Biochemistry Imo State University Owerri Nigeria; ^9^ Human and Animal Physiology Group Wageningen University and Research Wageningen the Netherlands; ^10^ German Research Center for Environmental Health Helmholtz Zentrum München Neuherberg Germany; ^11^ Abteilung für Nephrologie, Klinikum Rechts der Isar der Technischen Universität München München Germany; ^12^ Islet Biology Laboratory Centre for Biomolecular Interactions, University of Bremen Bremen Germany

**Keywords:** diabetes mellitus, diabetic nephropathy, end‐stage kidney disease, kidney replacement therapy, microvascular complication, phytochemistry

## Abstract

Diabetic kidney disease (DKD) is a severe complication of diabetes that presents as progressive kidney dysfunction and is the primary cause of end‐stage kidney failure. Despite therapeutic advances, including the use of angiotensin‐converting enzyme inhibitors, angiotensin receptor blockers, sodium‐glucose co‐transporter‐2 inhibitors, glucagon‐like peptide‐1 agonists, and non‐steroidal mineralocorticoid receptor antagonists, managing DKD remains challenging. Current therapies mainly focus on glycemic control, hypertension management, and albumin reduction to mitigate kidney damage. Nevertheless, these approaches often fail to halt disease progression or restore renal function. There is therefore an urgent need for therapies with safer profiles that can be used singly to target the disease's underlying pathophysiology or integrated into traditional care. Polyphenols possess biological properties capable of addressing these unmet needs by targeting multiple underlying mechanisms involved in DKD pathogenesis. The literature search occurred between September 2024 and April 2025, with most articles sourced from the last 5 years. This review explores polyphenol classes that have demonstrated nephroprotective effects in vitro, in vivo, and in clinical trials. It also highlights the interconnected multi‐pathways and molecular mediators potentially regulated by specific polyphenols for kidney function improvement. This multi‐target therapeutic approach is especially beneficial for DKD, where several metabolic dysfunctions underlie its pathogenesis. Identifying polyphenols as a therapeutic option could lead to integrative patient care that embraces the strengths of conventional medicine and phytomedicine for better disease management and outcomes. There is therefore a need for more clinical trials to assess polyphenols' safety and efficacy in managing DKD.

## Graphical abstract

This review shows evidence from preclinical and clinical studies, supporting the therapeutic potential of polyphenols for diabetic kidney disease (DKD). This is due to their diverse bioactive properties and ability to intervene in intricate mechanisms of the disease. Because DKD involves complex signaling mechanisms, polyphenols' ability to act synergistically and simultaneously on multiple targets makes them suitable therapeutic agents with a safe profile. Integrating them with conventional treatments may provide a more holistic and effective approach to managing DKD.

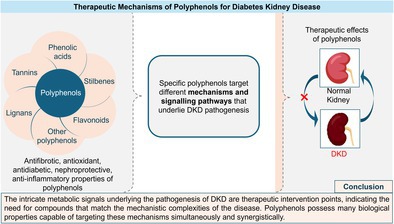

## 1 Introduction

Several underlying mechanisms are involved in the pathogenesis of diabetes and its complications (Zaghloul et al. 2022). As such, therapeutic intervention for diabetes and its complications should be targeted at the mechanisms involved (Esienanwan Esien Efiong et al. 2024; Wasana et al. 2021). Of the many complications associated with diabetes, diabetic kidney disease (DKD) is one of the most devastating and is one of the main causes of morbidity and mortality in patients with diabetes.

Despite the severity of the disease, there are limited therapeutic options that directly target its pathophysiology synergistically and simultaneously. Elevated glucose levels, blood pressure (Climie et al. 2019; Lin, Ma, and Wang 2022), and control of proteinuria are primarily addressed to slow the progression of renal damage (Ma et al. 2021). However, it is challenging to reverse progressive kidney damage due to its complicated pathogenesis.

As chronic hyperglycemia is the primary cause of renal failure, current therapy naturally focuses on the restoration of normoglycemia and does not include primary protection for the kidney. Insulin is the therapy for people with type 1 diabetes. Oral antidiabetic medication is the primary therapy for type 2 diabetes (T2D), although insulin can also be administered to people with severe insulin‐dependent T2D. Oral antidiabetic agents like biguanides, alpha‐glucosidase inhibitors, aldose reductase inhibitors, thiazolidinediones, sulfonylureas, insulin‐like growth factor, glucagon‐like peptide‐1 receptor agonists (Martin and Docherty 2024), amylin analogues, selective sodium‐glucose cotransporter‐2 (SGLT‐2) inhibitors, and dipeptidyl peptidase‐IV inhibitors act through different mechanisms (Adinortey et al. 2019; Elkhalifa et al. 2024; Niisato and Marunaka 2023; Omale et al. 2023; Utami et al. 2023; Yikna and Yehualashet 2021).

Although classical medications can improve glycemic control, they are associated with side effects (Dirir et al. 2022; Iwara et al. 2013; Pereira et al. 2019; Yang et al. 2020), which reduce their effectiveness and compliance rates. Standard treatments for reducing proteinuria and delaying the decline of renal function include renin–angiotensin–aldosterone system (RAAS) inhibitors like angiotensin‐converting enzyme inhibitors (ACEI) and angiotensin receptor blockers (ARBs) (Elendu et al. 2023; Samsu 2021). However, these drugs often exhibit limitations in achieving comprehensive kidney protection and are associated with adverse effects. Indeed, the use of dual renin–angiotensin–aldosterone system blockers is associated with hyperkalemia and cardiorenal events (Mallik and Chowdhury 2022; Selby and Taal 2020). Other side effects are fatigue, dizziness, dysgeusia, nausea, diarrhea, constipation, cough, proteinuria, oliguria, neutropenia, hypotension, and angioedema (linked to use of enalapril, captopril, and lisinopril) (Chakraborty and Roy 2021), dizziness, syncope, and weakness (Dehdashtian et al. 2020), especially as dual treatments (Lin, Ma, and Wang 2022). This limits their use for certain patients.

Inhibitors of SGLT2 reduce cardiovascular and renal events in patients with diabetes, including those with nephropathy (Bayne et al. 2023; Pelle et al. 2022; Selby and Taal 2020; Tang et al. 2022). Nevertheless, side effects associated with this treatment include glucosuria and osmotic diuresis (Cao et al. 2019). Despite the benefits of these inhibitors, deterioration of renal function is delayed but not halted (Horton and Barrett 2021; Wang, Wang, Liu, and Lan 2021; Yamazaki et al. 2021). In addition, one of the most common adverse events resulting from the use of some SGLTs, such as canagliflozin and dapagliflozin, is genital mycotic infections, which may often occur early in the treatment (Mallik and Chowdhury 2022) but can be controlled during chronic therapy. Another downside to the use of SGLT2 is the risk of diabetic ketoacidosis (Chen et al. 2022).

The limitations of these inhibitors suggest that additional mechanisms such as oxidative stress (OS), fibrosis, lipotoxicity, and inflammation might be major factors linked to the progression of DKD (Demir et al. 2021; Ma et al. 2021; Rayego‐Mateos et al. 2020). Hence, in addition to tight control of blood glucose and blood pressure, the direction of anti‐DKD agent research should be targeted at multiple mechanisms related to the disease. These include interrupting cell signaling pathways, targeting enzymes, reducing extracellular matrix (ECM) aggregation, and all other mechanisms underlying the pathogenesis of the disease (Dagar et al. 2021; Halim and Halim 2019; Jin et al. 2023; Kato and Natarajan 2015; Pradeep et al. 2019; Sankrityayan et al. 2019; Shen et al. 2019; Zahari Sham et al. 2022; Tang et al. 2021; Tziastoudi et al. 2021; Wang et al. 2020). Our recent work highlighted mechanisms, pathways, and mediators that are interconnected to drive the onset and pathogenesis of DKD (Efiong et al. 2025). Drugs should preferably act in a multifaceted way, allowing them to limit ECM synthesis and facilitate its disintegration (Lin, Ma, and Wang 2022).

Despite the significant burden of kidney diseases, effective therapeutic options are few, increasing mortality rates among patients. Thus, there is a need for novel drugs with proven safety and efficacy to treat DKD and other renal illnesses more efficiently (Elendu et al. 2023; Ma et al. 2021).

## 2 Current Perspectives on Phytomedicine for Diabetes Kidney Disease

Currently, research endeavors are focused on elucidating novel molecular signaling pathways (Advani 2020; Szostak et al. 2023) and developing advanced, multifactorial, safe, and effective therapeutic interventions (Prasanna and Parimala Devi 2011; Jin et al. 2023; Skalli et al. 2019; Wang et al. 2020; Wasana et al. 2021). Recently, complementary and alternative medicines from natural products have provided safe alternatives to synthetic medicine (Chakraborty and Roy 2021). There is documentation by the World Health Organization (WHO) on the use of traditional and complementary medicine from different countries for treating various diseases (World Health Organization 2013, 2019). WHO reported that a large part of the world relies on plant products as a primary source of health care (Monalisa and Perbawati 2022; World Health Organization 2013; Tienda‐Vazquez et al. 2022). According to a report (Laha and Paul 2019), WHO highlighted 21,000 plants with medicinal purposes globally. WHO further estimated that 25% of the currently used drugs are of plant origin. Of the small‐molecule drugs developed over the past few decades, 5% originated from natural products, 27% were derivatives of natural products, and 30% were synthetic drugs inspired by natural products (Wasana et al. 2021). Estimates from the WHO also show that more than 1200 medicinal plant species are used globally in treating diabetes, mainly in developing nations, representing over 725 genera in 183 families (Yikna and Yehualashet 2021).

### 2.1 Phytomedicine and Its Multimodal Cascade of Activity in Diabetes and Diabetic Kidney Disease

Phytomedicine is affordable, accessible, and often has minimal side effects (Salehi et al. 2019; Shaito et al. 2020; Skalli et al. 2019). It targets different pharmacological pathways linked to diabetes and DKD, which makes it easy to treat different underlying mechanisms of both diseases at the same time (Dehdashtian et al. 2020). Some of the mechanisms targeted by phytoconstituents include antioxidant effects; restoration of the functional mass of β cells; reversal of insulin resistance (IR); improved insulin secretion; suppression of glucose digestion and absorption; and promotion of glucose utilization. Others include modulating enzymes like lipoprotein lipase, glucose‐6‐phosphatase, lactate dehydrogenase, and aldose reductase; and improvement of hyperlipidemia, renal function, and other diabetic complications (Dewanjee et al. 2020; Pereira et al. 2019; Unuofin and Lebelo 2020).

In addition, the renoprotective activity of medicinal plant extracts is achieved via the nuclear factor kappa‐light‐chain‐enhancer of activated B cells (NF‐κB) signaling pathway (Salehi et al. 2019), reduction of OS, suppression of inflammation, reduction of advanced glycation end‐products (AGEs) production, cell apoptosis, and tissue injury‐related protein expression (Putra et al. 2023). These multi‐targeted activities of bioactive principles align with the complex pathophysiology of DKD, making it a more comprehensive therapeutic option than single‐targeted intervention.

Exploring phytomedicine will, therefore, contribute to improved outcomes and create a more resilient defense against the progression of renal complications. Although many bioactive compounds in plants work additively, single compounds from plants still need to be delineated for a more specific, targeted approach to the therapy of DKD. Polyphenol is a large class of phytochemicals well‐known for its biological activity, including antidiabetic, anti‐inflammatory, antioxidant, and renoprotective effects. These biological activities position polyphenols as promising candidates for mitigating the pathogenesis of DKD, which necessitates further investigation into their interaction with the molecular mechanisms implicated in it.

### 2.2 Classification of Polyphenols and Their Mode of Action on DKD in Different Experimental Models

**FIGURE 1:**
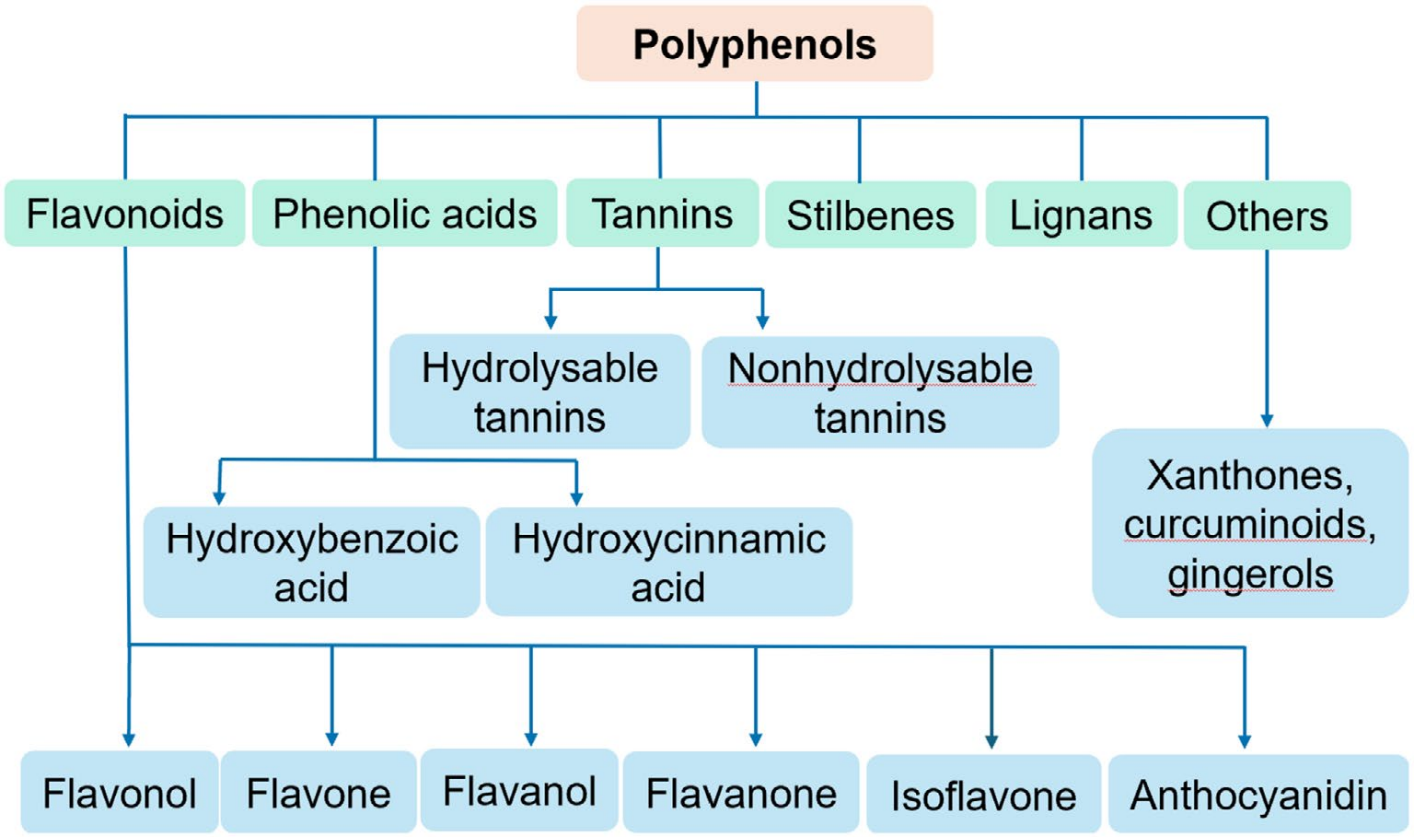
Classification of polyphenols. The structures were drawn using Kingdraw.

Polyphenols are the most abundant antioxidants in the human diet (Yang et al. 2020). They include over 8000 different molecules (Jin et al. 2023) with many biological effects (Efiong 2014). According to their chemical structure, they can be divided into four major groups: flavonoids, phenolic acids, stilbenes, and lignans (Jin et al. 2023) (Figure 1). In vivo, in vitro, and clinical trials confirm the biological role of polyphenols in the pathogenesis of DKD (Saad et al. 2022).

## 3 Classification of Flavonoids and Their Molecular Mechanisms of Action

Among several phytochemical classes, flavonoids have outstanding pharmacological effects (Adnan et al. 2021). They are a significant class of polyphenolic compounds (Chakraborty and Roy 2021) including flavonols, flavones, flavan‐3‐ols, isoflavones, flavonones, and anthocyanins with low molecular weight (Adnan et al. 2021). They represent a large group of plant secondary metabolites present in many vegetables, fruits, and herbs (Salehi et al. 2019). They are chemically characterized by two phenyl rings bound to a heterocyclic ring. The presence of aromatic rings and hydroxyl groups in the structure of flavonoids, especially the “catechol” moieties, with two phenolic OH‐groups in ortho position from each other accounts for their use as natural antioxidants. Many studies show the efficacy of flavonoids in improving diabetes‐related complications (Pereira et al. 2019), such as lowering blood glucose and lipids, improving kidney function, inhibiting mesangial matrix expansion, and glomerular hypertrophy (Yang, Xue, et al. 2019; Yikna and Yehualashet 2021). Phenolic compounds, including flavonoids, play an important role in free radical scavenging (Aryal et al. 2021). They enhance β‐cell survival against high glucose (HG), high proinflammatory cytokines, and lipids by inhibiting NF‐κB activation, promoting PI3K/protein kinase B (AKT) signaling, inhibiting nitric oxide synthesis, and OS reduction. Flavonoids restore the secretory function of β‐cells via protein kinase C (PKC), PKA, phospholipase C, and cyclic adenosine monophosphate (cAMP) (Dewanjee et al. 2020).

### 3.1 Flavan‐3‐ols

#### 3.1.1 Catechins

**FIGURE 2:**
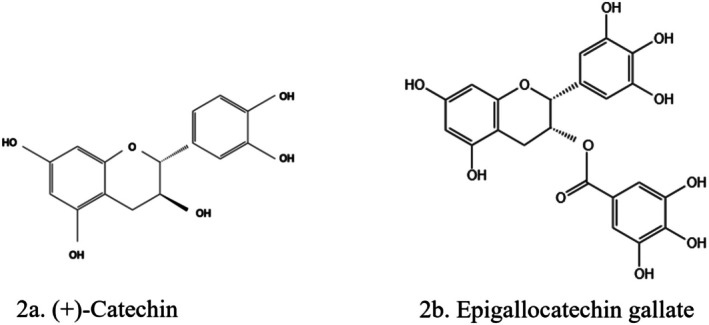
Structures of flavan‐3‐ols: (a) (+)‐Catechin, (b) epigallocatechin‐3‐gallate. The structures were drawn using Kingdraw.

The class of flavonoids called catechins includes, a.o. epicatechin, epigallocatechin, epicatechin gallate, catechin, and epigallocatechin‐3‐gallate (EGCG). They are the active constituents of cocoa products and *Camellia sinensis* (green tea) (Avila‐Carrasco et al. 2021). The main bioactive catechin in green tea is EGCG (Kaabi 2022) (Figure 2). Green tea is one of the most popular beverages worldwide, with a high concentration of polyphenols (Basu et al. 2023). They have antioxidant effects and enhance the activities of glutathione (GSH) *S*‐transferase, superoxide dismutase (SOD), and catalase (CAT) (Salehi et al. 2019). Green tea prevents diabetes, hypertension, renal injury, and OS. It is effective in reducing obesity and obesity‐related inflammation (Saad et al. 2022). EGCG promotes insulin secretion, protects the islets of Langerhans, reduces insulin tolerance, and generates glucose from free fatty acids and lipids (Unuofin and Lebelo 2020). Catechin found in *C. sinensis* inhibited AGEs formation and lowered pro‐inflammatory cytokines interleukin‐1beta (IL‐1β) and tumor necrosis factor alpha (TNFα) levels by methylglyoxal trapping (Parveen et al. 2018). (+)‐Catechin exerted a renoprotective effect in a diabetic rat model (Chennasamudram et al. 2012) and ameliorated DKD by trapping methylglyoxal in T2D mice (Zhu et al. 2014). Multiple experimental models, including human studies, confirmed catechins' antiobesity potentials (Basu et al. 2023).

The use of catechins in treating and preventing kidney disease is often related to inflammation and OS, by direct inhibition of stress or stimulus‐induced reactive oxygen species (ROS) overproduction (Patane et al. 2023). Additionally, they could impact the nuclear factor erythroid 2‐related factor 2 (Nrf2)‐Keap1‐Cul‐3 complex, causing translocation of free Nrf2 into the nucleus. Nrf2 then binds to the antioxidant response element in the promoter region of cytoprotective genes and genes encoding antioxidant enzymes (Avila‐Carrasco et al. 2021). A study (Liao et al. 2022) showed that EGCG improved renal fibrosis by inhibiting transforming growth factor‐beta 1 (TGF‐β)/mothers against decapentaplegic homolog 3 (Smad3) pathway in diabetic mice. The anti‐inflammatory effect of EGCG in various chronic kidney disease models was reported (Kaabi 2022).

### 3.2 Flavanones

#### 3.2.1 Naringenin

**FIGURE 3:**
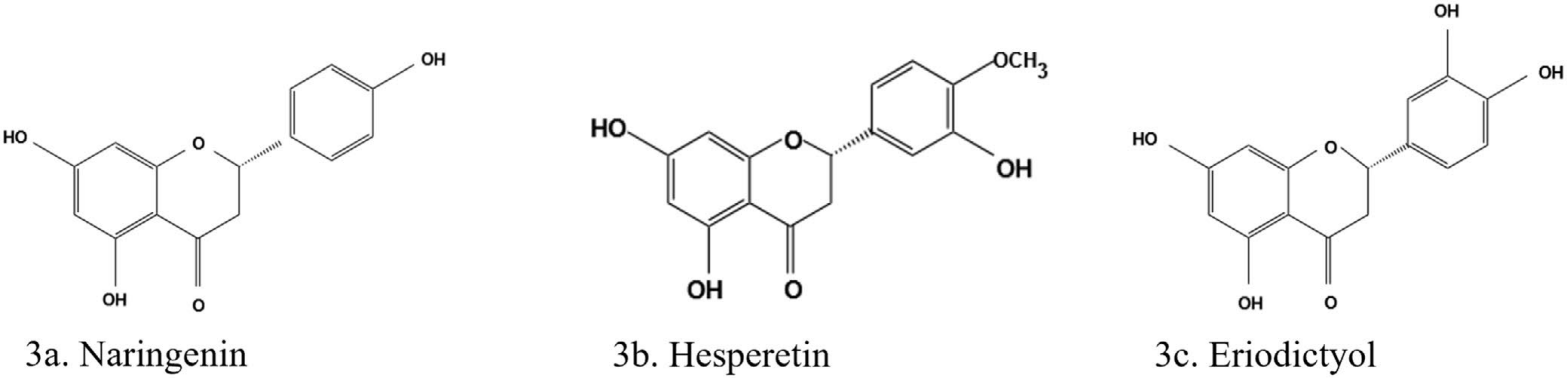
Structures of flavanones. (a) Naringenin, (b) hesperetin, (c) eriodictyol. The structures were drawn using Kingdraw.

Naringenin (4′,5,7‐Trihydroxyflavanone) (Figure 3a) is a flavonoid that is mostly found in citrus fruits (Hu et al. 2021) and has very strong antioxidant properties. It reduces glucose adsorption in the intestinal brush border (Salehi et al. 2019) and has strong antidiabetic properties (Abu‐Odeh and Talib 2021; Wang et al. 2022; Yikna and Yehualashet 2021). This flavonoid was reported to cause reduced renal glucose reabsorption and increased muscle and fat tissues glucose uptakes. Naringenin reduced triacylglyceride synthesis and gluconeogenesis in hepatocytes, resulting in the attenuation of hyperglycemia and dyslipidemia (Salehi et al. 2019; Yang et al. 2020). It enhanced insulin receptor substrate (IRS) 1, glucose transporter 1 and 3 levels; improved the signaling of proliferator‐activated receptor gamma (PPARγ) and adenosine monophosphate‐activated protein kinase (AMPK); hence, improving insulin sensitivity (Unuofin and Lebelo 2020). Naringenin also improved the function of β‐cells by turning on genes for glucose transporter 2, IRS, AKT, duodenal and pancreatic homeobox 1, B‐cell lymphoma 2, and heat shock protein 70/90. Naringenin also inhibited β‐cell loss by downregulating proapoptotic genes—Bcl‐2‐associated X protein (Bax), caspase 3, and acetyl‐CoA carboxylase (Dewanjee et al. 2020).

Naringenin upregulated the expression of 20‐hydroxyeicosatetraenoic acid, cytochrome P450, and peroxisome PPARs in renal tubular epithelial cells (NRK‐52E) in a dose‐dependent manner (Hu et al. 2021). It also functions as a Smad3 inhibitor (Wang, Wang, Liu, and Lan 2021) of AMPK. Thus, the protective properties of naringenin in DKD are via suppression of NADPH oxidase 4 (NOX4) expression (Kaabi 2022). Naringenin attenuated DKD through its anti‐inflammatory and antifibrotic activities by decreasing the expression of IL‐1β, IL‐6, fibronectin, type IV collagen, and TGF‐β1 (Salehi et al. 2019).

#### 3.2.2 Hesperetin

Hesperidin (hesperetin‐7‐O‐rutinoside) (Figure 3b) is a flavanone glycoside found in high concentrations in different parts of citrus fruits, peppermint, and other plants (Mirzaei et al. 2023; Novotna et al. 2023). However, it shows more bioactivity and reactivity in its aglycone form (hesperetin, a flavanone (Rasania and Sharan 2023)). The conversion of hesperidin to hesperetin takes place during passage through the gastrointestinal tract (Mirzaei et al. 2023; Ruviaro et al. 2020; Yang et al. 2023). Hesperidin and hesperetin have antidiabetic (Naz et al. 2023; Parasuraman and Thinagaran 2023), antihypertensive, antiallergic, radioprotective, and immunomodulatory effects (Chukwuma 2024; Kakadiya et al. 2010).

Hesperetin upregulates the level of glyoxalase 1, blocks the AGE/RAGE axis, suppresses related inflammation (Rayego‐Mateos et al. 2020), increases the levels of Nrf2 and p‐Nrf2, and upregulates the expression of the Nrf2/antioxidant response element signal transduction target gene γ‐glutamyl half cystine synthase by activating the Nrf2/antioxidant response element pathway (Putra et al. 2023). Hesperetin also suppressed DKD by inhibiting the TGF‐β1/integrin‐linked kinase (ILK) AKT pathway in streptozotocin (STZ)‐induced diabetic rats. This indicates that the expression of TGF‐β1 and its downstream effectors AKT and ILK was inhibited, thereby alleviating DKD (Hu et al. 2021). Other renoprotective mechanisms of hesperetin include blocking of epithelial–mesenchymal transition (EMT) in TGF‐β‐treated podocytes by regulation of the mechanistic target of rapamycin (mTOR) pathway (Kang et al. 2020).

In a dietary intervention that involved 31 people with T2D, a 6‐week daily administration of hesperetin (500 mg) significantly lowered blood pressure, inflammatory markers (IL‐6, TNF‐α, and high‐sensitivity C‐reactive protein), and elevated serum total antioxidant capacity (Jin and Arroo 2023). Hesperetin also inhibited OS, HG‐induced cell proliferation, and the expression of extracellular matrix in glomerular mesangial cells (MCs), reducing the level of ROS and malondialdehyde (MDA) and inhibiting the expression of fibronectin and type IV collagen (Hu et al. 2021; Rasania and Sharan 2023).

Renoprotective activities of hesperidin are modulation of TGF‐β1 and oxidative DNA damage (Kandemir et al. 2017) and modulation of matrix metallopeptidase 9 and apoptosis (Hassan et al. 2023).

#### 3.2.3 Eriodictyol

Eriodictyol (S‐3′,4′,5,7‐tetrahydroxyflavanone) (Figure 3c), a flavonoid found in fruits and vegetables, especially peanuts and citrus (Hussain et al. 2020; Kaabi 2022), has positive effects on obesity and diabetes (Hussain et al. 2020). Eriodictyol is one of the most abundant flavonoids in *Eriodictyon californicum* (Kong et al. 2021). It acts therapeutically against DKD (Jin and Arroo 2023; Rasania and Sharan 2023). It upregulated messenger ribonucleic acid (mRNA) expression of adipocyte‐specific fatty acid binding protein, PPARγ2, and protein of PPARγ2 in differentiated 3 T3‐L1 adipocytes. It reactivated AKT in HG‐induced insulin‐resistant HepG2 cells (Vinayagam and Xu 2015). Eriodictyol protects against diabetic retinopathy (Kong et al. 2021) and significantly lowers retinal TNFα, intercellular adhesion molecule 1, vascular endothelial growth factor (VEGF), and endothelial nitric oxide synthase.

Treatment with eriodictyol significantly suppressed diabetes‐related lipid peroxidation. It also protects MCs from mercury stimulation by inhibiting the activity of the AKT–NF‐κB pathway, suppressing the production of extracellular matrix proteins, and secreting inflammatory cytokines (Hu et al. 2021). Eriodictyol also protected MCs from high glucose (HG) stimulation via mitigation of the AKT/NF‐κB signaling pathway (Bai et al. 2019; Hu et al. 2023; Rasania and Sharan 2023). The capacity of eriodictyol to protect RGC‐5 cells against HG‐induced damage is by controlling the Nrf2/heme oxygenase‐1 (HO‐1) signaling pathway (Kaabi 2022). Eriodictyol obstructs NOX2 and NOX4 production, shortening ROS and MDA synthesis, and protecting MCs from HG stimulation by inhibiting collagen, fibronectin, type IV collagen, and ECM synthesis (Jin et al. 2023). Studies (AlTamimi et al. 2023; Modepalli Poojitha et al. 2023) confirmed that the renoprotective impact of eriodictyol was via antioxidant and anti‐inflammatory mechanisms by Nrf2 activation.

### 3.3 Flavones

#### 3.3.1 Luteolin

**FIGURE 4:**
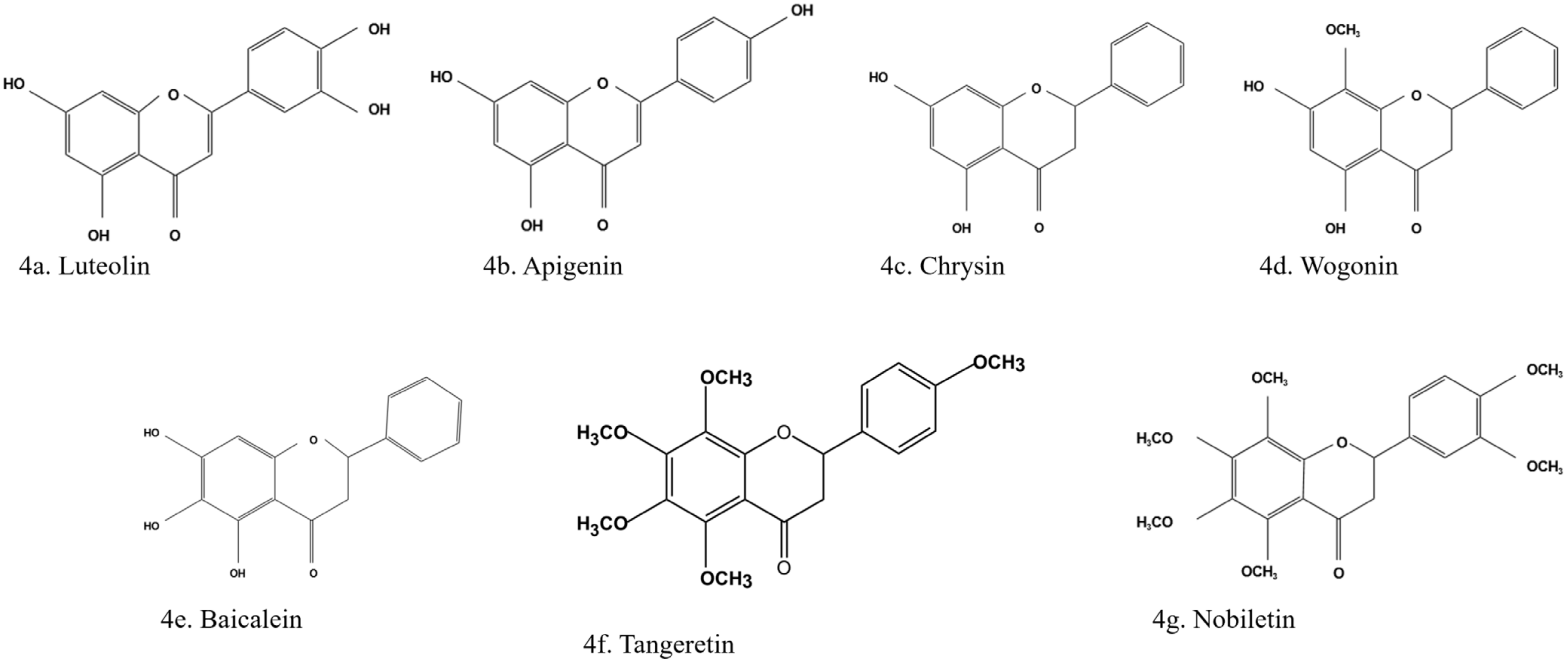
Structures of flavones. (a) Luteolin, (b) apigenin, (c) chrysin, (d) Wogonin, (e) baicalein, (f) tangeretin, (g) nobiletin. The structures were drawn using Kingdraw.

Luteolin (5,7,3′,4′‐Tetrahydroxyflavone) (Figure 4a) is a natural flavonoid common in many fruits and vegetables (Liu, Yuan, et al. 2022), such as honeysuckle, *Nepeta chrysanthemum* (catmint), carrots, sweet peppers, peppers, celery, groundnuts (Hu et al. 2021), cabbage, apple skins, parsley, onion, broccoli, and *Chrysanthemum* flowers (Menati et al. 2020). A study showed that luteolin inhibited α‐glucosidase better than acarbose, a standard drug. The study's findings showed that luteolin further improved hepatic insulin sensitivity by suppressing the expression of sterol regulatory element‐binding transcription protein 1 that modulates IRS‐2 expression through its negative feedback and gluconeogenesis (Adinortey et al. 2019). Luteolin ameliorated cardiac failure in type 1 diabetes cardiomyopathy by inhibiting TGF‐β1/Smad signaling (Salehi et al. 2019). It also protected the filtration function of the glomerular basement membrane by upregulating podocin (NPHS2 protein) expression, which strikingly suppressed apoptosis and deletion and fusion of STZ‐induced diabetic rat podocytes in high‐glucose conditions. Luteolin inhibited the anti‐inflammatory response and OS by suppressing the activity of the signal transducer and activator of transcription 3 (STAT3) pathway, thereby reducing renal fibrosis and delaying the progression of DKD (Hu et al. 2021).

Luteolin attenuated HG‐induced podocyte damage via suppression of the NOD‐like receptor family pyrin domain‐containing 3 (NLRP3) inflammasome pathway. It suppressed inflammatory response and OS by inhibiting the STAT3 pathway (Putra et al. 2023). Luteolin further modulates molecular targets, like transcription factor NF‐κB, activation of cytokines (IL‐1, IL‐6, TNFα, and chemokines), protein‐1, and inducible nitric oxide synthase inhibitor (Menati et al. 2020). Luteolin obtained from *Chrysanthemum indicum* exhibited aldose‐reductase inhibitory activity and prevented the morphological destruction of the kidneys in diabetic rats caused by increased OS via the polyol pathway (Parveen et al. 2018). It also prevented DKD through increased heme oxygenase‐1 expression, antioxidant status (Jin et al. 2023), and reduced MDA content (Parveen et al. 2018).

#### 3.3.2 Apigenin

Apigenin (4′,5,7‐Trihydroxyflavone) (Figure 4b) is abundantly present in oranges, onions, parsley (Hu et al. 2021), celery, oregano, chamomile, thyme, basil, tea, beer, nuts, soy, and many other plants (Menati et al. 2020). Apigenin is a known antidiabetic agent (Abu‐Odeh and Talib 2021) that decreased the release of TNF‐α, IL‐1β, and IL‐6 via PI3K/AKT in HK‐2 tubular cells under HG conditions (Jin et al. 2023; Rayego‐Mateos et al. 2020). It alleviated renal dysfunction, fibrosis, and OS by inhibiting fibronectin, TGF‐β1, and type IV collagen. Apigenin significantly suppresses the activation of the downstream inflammatory mitogen‐activated protein kinase (MAPK) pathway by lowering the expression of IL‐6, TNF‐α, and NF‐κB and reducing the expression of apoptotic proteins Bax and caspase‐3.

Apigenin decreased the apoptosis of renal tubular epithelial cells by inhibiting OS and increasing the expression of Nrf2 and heme oxygenase‐1 (HO‐1) in a DKD model (Hu et al. 2021; Kaabi 2022). A study (Menati et al. 2020) showed that apigenin mitigated STZ‐induced nephropathy in rats by regulating OS and reducing kidney fibrosis by TGF‐β1‐(MAPKs)‐fibronectin and MAPK/NF‐κB/TNFα pathways.

#### 3.3.3 Chrysin

Chrysin (5,7‐Dihydroxyflavone) (Figure 4c), a flavone predominantly found in *Passiflora caerulea* , *Passiflora incarnata* , and *Oroxylum indicum*, suppressed fibronectin, TGF‐β1, and collagen‐IV protein expressions in renal tissues. It also reduced the serum levels of IL‐1β and IL‐6 (Salehi et al. 2019). Chrysin reduced IR, improved inflammation, OS, and liver damage through induction of AMPK signaling, which prevented IR in HepG2 cells by enhancing IRS‐1 overexpression and induction of AKT (Entezari et al. 2022). It also alleviates IR‐related vascular complications via the PPAR‐c‐dependent pathway (Pan et al. 2022). A study documented the effect of chrysin from different diabetes experimental models to include inhibition of the TNF‐α pathway, which resulted in reduced secretion of proinflammatory cytokines, reduction of blood glucose, OS, and improvement of memory function (Vinayagam and Xu 2015). Chrysin inhibited diabetes‐induced kidney tubulointerstitial fibrosis by suppressing EMT. It also ameliorated podocyte injury via inhibition of the PERK/eukaryotic initiation factor 2α (eIF2α)/activating transcription factor and C/EBP homologous protein (CHOP) pathways involved in endoplasmic reticulum (ER) stress. Chrysin inhibits kidney fibrosis related to AGEs in renal MCs and diabetic kidneys by reducing the accumulation of matrix proteins and myofibroblast‐like cells (Menati et al. 2020). Chrysin's effect against DKD is reportedly through its anti‐inflammatory effects in renal tissues by targeting the TNF‐α pathway (Jin and Arroo 2023).

#### 3.3.4 Wogonin

Wogonin (5,7‐dihydroxy‐8‐methoxyflavone) (Figure 4d) is isolated from *Scutellaria baicalensis* Georgi. It has positive activities on insulin sensitivity, blood glucose, and lipid metabolism through selective AMPK and PPARα signals (Vinayagam and Xu 2015). Its antiadipogenic property in a mouse model for non‐insulin‐dependent diabetes (C57BLKS/JLeprdb/Leprdb) was reported (Hussain et al. 2020). It has proven to have a therapeutic effect on DKD by preventing OS and inflammatory processes. Wogonin alleviates renal inflammation and fibrosis in DKD by inhibiting the (TGF‐β1)/Smad3 and NF‐κB signaling pathways (Zheng et al. 2020). Another study documented the repression of the PI3K/AKT/NF‐κB pathway by wogonin via attenuation of kidney tubular epithelial injury in DKD (Gao et al. 2022; Xu et al. 2024).

#### 3.3.5 Baicalein

Baicalein (5,6,7‐Trihydroxyflavone) (Figure 4e) is a flavonoid isolated from the roots of *Scutelleria baicalensis* and *S. lateriflora* (Ahad et al. 2014). The antioxidant effects of baicalein isolated from *Oroxylum indicum* and *S. baicalensis* have also been reported (Chen et al. 2022; Salehi et al. 2019). The phytoconstituent has antidiabetic activity via the activation of AMPK, resulting in reduced IR by phosphorylating AKT and IRS‐1 and inducing dephosphorylation of ERK, NF‐κB, and c‐Jun N‐terminal kinase (JNK) (Unuofin and Lebelo 2020). Baicalein enhances the action of metformin by reducing the circulation of branched‐chain amino acids, thereby improving insulin sensitivity via normalizing de novo lipogenesis and mTORC1/p70S6K/IRS1 signaling (Xing et al. 2024). It also reduces inflammation and OS in DKD by modulating MAPK and Nrf2 signaling (Kaabi 2022). Baicalein prevented DKD development in high‐fat diabetes/STZ‐induced T2D rats via its antioxidant, antihyperglycemic, and anti‐inflammatory activities by suppressing NF‐κB (Ahad et al. 2014). In a clinical study, baicalein improved renal function in patients with DKD and delayed the progress of the disease through the polyol pathway, antioxidant, and anti‐inflammatory mechanisms (Yang, Kan, et al. 2019). Other renoprotective mechanisms of baicalein are through prevention of HG‐induced podocyte apoptosis (Li et al. 2020).

Baicalin is a glucuronic acid derivative of baicalein. Upon oral intake, baicalin is metabolized to release baicalein. While in vitro studies on baicalin may demonstrate renoprotective effects, in vivo and clinical studies may attribute its activity to baicalein. In vitro tests on HG‐induced podocytes showed that baicalin protected against DKD (Jin et al. 2023) by raising the level of sirtuin (SIRT) 1 and blocking the NF‐κB pathway (Modepalli Poojitha et al. 2023).

#### 3.3.6 Tangeretin

Tangeretin (4′,5,6,7,8‐Pentamethoxyflavone) (Figure 4f) is a polymethoxylated flavonoid extracted from orange peel. It stimulates the activation of AMPK (88), which is the mechanism associated with its anti‐inflammatory activity (Vinayagam and Xu 2015). In STZ‐induced diabetic rats, it lowered plasma glucose, increased insulin and hemoglobin, and modulated hepatic enzyme activities (Yang et al. 2020). By stopping EMT, it lowered the damage and fibrosis caused by glucose OS and hypoxia in rats. This resulted in a decrease in the expression levels of the epithelial markers E‐cadherin and P‐cadherin, and an increase in the expression of podocyte slit diaphragm protein. Tangeretin inhibits apoptosis in human glomerular MCs by inhibiting the extracellular signal‐regulated kinase pathway (Hu et al. 2023).

#### 3.3.7 Nobiletin

Nobiletin (5,6,7,8,3′,4′‐Hexamethoxyflavone) (Figure 4g) is a polymethoxyflavone present in citrus fruits like *Citrus depressa* (shiikuwasha), *Citrus sinensis* (oranges), and *Citrus limon* (lemons) (Menati et al. 2020). Nobiletin has antiapoptotic (Zhang, Cheng, et al. 2023), antioxidant (Afzal et al. 2023), anti‐inflammatory, blood pressure, and cholesterol‐lowering activities. It can lower IR, regulate blood glucose, protect β‐cells, and improve acute kidney injury (Wang, Wang, Yang, et al. 2021). It also has antiferroptotic and antifibrotic effects, which alleviate the progression of DKD (Song et al. 2023). In a mice experimental model, nobiletin was effective at preventing InR, dyslipidemia, obesity, and hepatic steatosis (Rahmadhanita et al. 2023). Through the STAT3/NFκB pathway, nobiletin stopped the inflammation and ECM buildup caused by high glucose levels in human MCs (Menati et al. 2020).

### 3.4 Flavonols

#### 3.4.1 Quercetin

**FIGURE 5:**
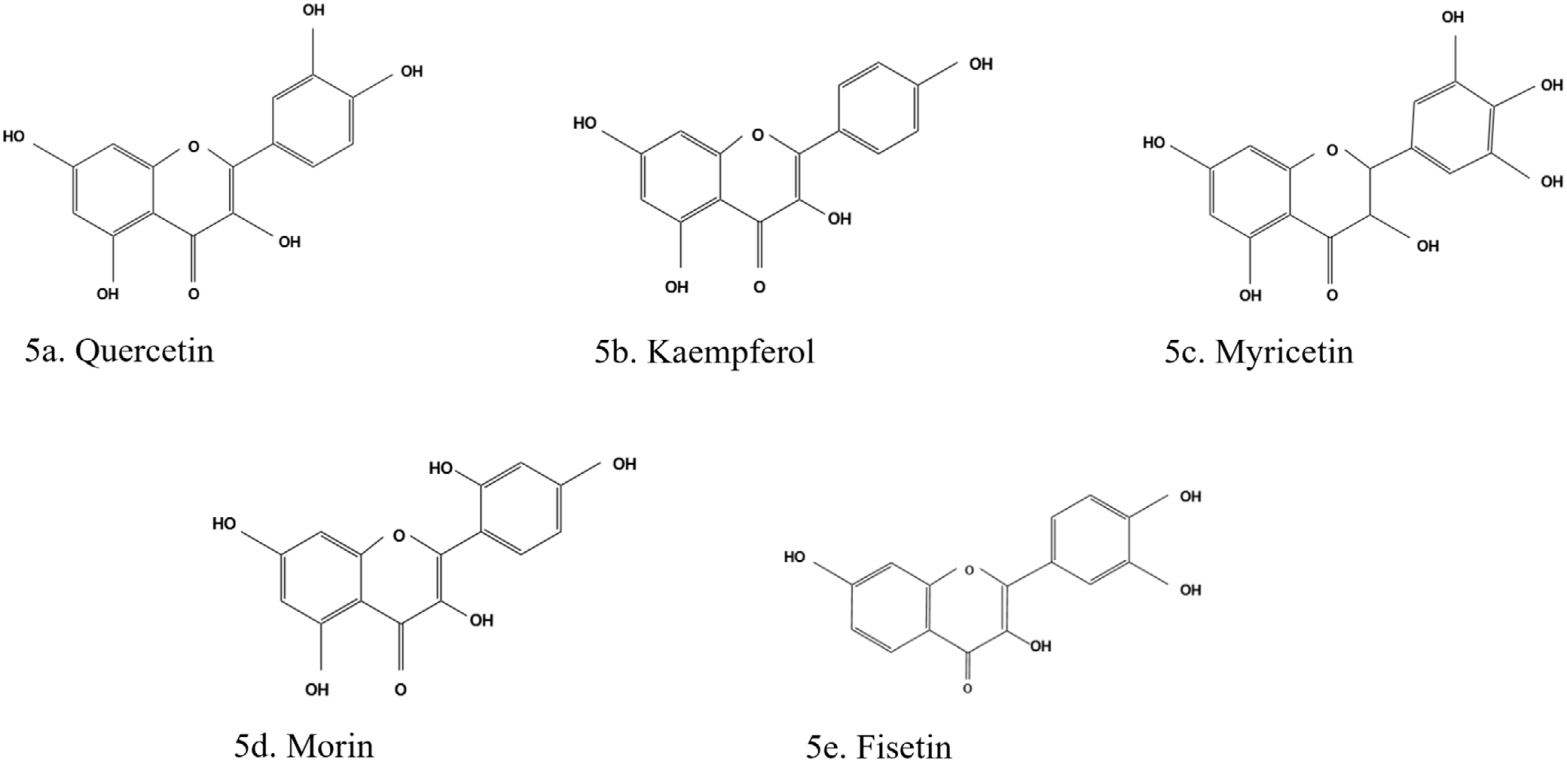
Structure of flavonols. (a) Quercetin, (b) kaempferol, (c) myricetin, (d) morin, (e) fisetin. The structures were drawn using Kingdraw.

Quercetin (3,3′,4′,5,7‐Pentahydroxyflavone) (Figure 5a) is a flavonol found in blackcurrants, apples, chokeberries, cherries (Parveen et al. 2018), stems and leaves of buckwheat, hawthorn, sea buckthorn, onion, and many other plants. It exists mostly in the form of glycosides, such as rutin and hyperoside. Quercetin, alongside some organosulphur compounds like S‐methylcysteine sulphoxide, allyl propyl disulphide oxide (dipropyl disulphide oxide), and S‐allyl cysteine sulphoxide in onions ( *Allium cepa* ), has good glucose and lipid regulatory activities. Quercetin reduces the risk of kidney dysfunction and cardiovascular diseases. It has antioxidant, antitumor, antiviral, and anti‐inflammatory effects (Dehdashtian et al. 2020; Jin et al. 2023; Sun et al. 2022). Quercetin reduced the levels of GSH, CAT, and high‐density cholesterol in experimental studies (Parveen et al. 2018).

Quercetin and other active components were reported to improve the activities of liver hexokinase, glucose‐6‐phosphatase, and 3‐hydroxy‐3‐methyl‐glutaryl‐CoA reductase (Adinortey et al. 2019; Unuofin and Lebelo 2020). Quercetin and the uric acid‐lowering drug allopurinol were reported to reduce the expression of the inflammatory markers (IL‐1β and IL‐18). They also ameliorated kidney damage in an STZ‐rat model (Rayego‐Mateos et al. 2020). Quercetin lowered the cell percentage in the G (0)/G (1) phase, as well as the levels of laminin, Smad 2/3 expression, type IV collagen, and TGF‐β (1) expression. It also activated the AKT/cAMP response element‐binding protein signal (Salehi et al. 2019). Quercetin exhibits renoprotective action by reducing inducible nitric oxide synthase and inhibiting NF‐κB translocation. It inhibited the overexpression of TGF‐β1 and CTGF in STZ‐induced diabetic rats and exerted its renoprotective effect via inhibition of renal fibrosis and the mTORC1/P70s6k signaling‐mediated renal tubular EMT (Adnan et al. 2021; Lei et al. 2019).

The study (Lei et al. 2019) reported that quercetin inhibited MC proliferation in HG‐treated mouse glomerular MCs and in DKD, resulting in improved renal function, renal fibrosis, and lipid levels. According to the study, the inactivation of the Hippo pathway was involved in HG‐induced MC proliferation, and quercetin played an antiproliferation role by reactivating the Hippo pathway (Sun et al. 2022). The renoprotective effect of quercetin is via reduction of CTGF expression, TGF‐β1 (Putra et al. 2023), lipid peroxidation, and ROS production. Thus, it contributes to the reduction of mesangial matrix expansion in glomerulosclerosis, capillary, and tubular basal membrane thickening. The renoprotective effect of quercetin in DKD also results from inhibition of renal tubular EMT, mediated by inhibition of mTORC1/p70S6K signaling pathways, which are associated with the inhibition of snail and twist transcriptional factors regulating E‐cadherin expression (Dehdashtian et al. 2020).

Quercetin improves the renal lipid droplet accumulation induced by diabetes via regulation of SCAP‐SREBP2‐low‐density lipoprotein receptors signaling, resulting in improved renal function, a normalized glomerulosclerosis index, and a kidney weight/body weight ratio. Also, quercetin inhibited lipid accumulation in diabetic rats' kidneys by suppressing the renal inflammasome component NLRP3. This led to reduced expression of caspase‐1, apoptosis‐associated speck‐like protein, IL‐1β, and IL‐18 (Dehdashtian et al. 2020). Quercetin considerably lowered TNF‐α, IL‐1β, MDA, urine albumin, blood urea nitrogen, urine protein, serum creatinine, and renal index in an experimental model (Kaabi 2022). It antagonized glucose fluctuation‐induced kidney injury by inhibiting aerobic glycolysis through the hypoxia‐inducible factor‐1 alpha (HIF‐1α)/miR‐210/iron–sulfur cluster assembly enzyme pathway in glomerular MCs (Chen et al. 2022). Quercetin further ameliorated renal fibrosis by upregulating the autophagy level mediated by beclin‐1 and downregulating snail1 expression (Putra et al. 2023).

Rutin, also called 3,3′,4′,5,7‐pentahydroxyflavone‐3‐rhamnoside or quercetin‐3‐*O‐rhamnoside*, is a flavonoid glycoside of quercetin abundant in tea and plants like apple, passionflower, and buckwheat (Hu et al. 2021). Its antidiabetic (Salehi et al. 2019), antioxidant, and anti‐inflammatory activities have been reported (Yang et al. 2020). Its renoprotective activities are via the RhoA/Rho‐associated coiled‐coil‐containing kinase signaling pathway, suppressing renal fibrosis in HG‐induced glomerular MCs (72), upregulating Janus Kinase‐2 (JAK2)/STAT3 and Nrf‐2/HO‐1 (Kaabi 2022), TGF‐β1/Smad/ECM, and TGF‐β1/CTGF/ECM signaling cascades (Parveen et al. 2018).

#### 3.4.2 Kaempferol

Kaempferol (Figure 5b), also called 3,4′,5,7‐Tetrahydroxyflavone, is a natural flavonol in tea, cruciferous vegetables, fruits, and many other plants (Hu et al. 2021). It has antioxidant, anti‐inflammatory, and lipolysis effects (Wang, Wang, Yang, et al. 2021). It promotes insulin sensitivity and preserves pancreatic β‐cell mass (Salehi et al. 2019). Kaempferol reduced OS in diabetic rats by activating the Nrf‐2/HO‐1/antioxidant axis (Putra et al. 2023). It reversed kidney damage in experimental animals due to its antioxidant and anti‐inflammatory effects, which resulted in decreased TNF receptor‐associated factor 6 levels. In RPTEC and NRK‐52E cells, kaempferol also reduced DKD (Kaabi 2022) by inhibiting the inflammatory signal caused by RhoA/Rho kinase, IL‐1β, and TNF‐α (Hu et al. 2021).

#### 3.4.3 Myricetin

Myricetin (3,5,7,3′,4′,5′‐Hexahydroxyflavone) (Figure 5c) was first isolated from the bark of the “myricetin” plant (Zhao et al. 2022). It is abundant in walnuts (Unuofin and Lebelo 2020), tea, wine produced from berries, vegetables, and several other plants (Hu et al. 2021). The antidiabetic activity of myricetin was documented (Al‐Abbasi and Kazmi 2023; Niisato and Marunaka 2023). Myricetin enhances IRS‐1‐related glucose transporter 4, PI3‐kinase transfer/movement, improvement of PPARα, and the suppression of SREBP hepatic expression (Unuofin and Lebelo 2020). Myricetin improved the activity of GSH peroxidase (GPX) and xanthine oxidase in kidney tissue; increased the expression of glycogen, insulin, glycogen synthase, and insulin signaling molecules, including glucose transporter type 2 and 4, insulin receptor substrate‐1 (IRS‐1), IRS‐2, and PKB. It upregulated SREBP‐1a, SREBP‐1c, SREBP‐2, VEGF, TGF‐β1, and PPAR‐α expression in DKD experimental animals, resulting in the amelioration of abnormal glucose and lipid metabolism and thus alleviating the progression of renal fibrosis (Hu et al. 2021).

Myricetin alleviated diabetes‐associated kidney injuries and dysfunction in an experimental model via induction of expression and translocation of Nrf2 and by inhibition of the IκBα/NF‐κB pathway (Yang, Wang, et al. 2019). Other mechanisms implicated in the renoprotection of myricetin include the upregulation of Nrf2/HO‐1 (Berkoz et al. 2024) and induction of M2 macrophage polarization to impede renal tubulointerstitial fibrosis in DKD through PI3K/AKT (Xu et al. 2024).

#### 3.4.4 Morin

Morin (3,5,7,2′,4′‐Pentahydroxyflavone) (Figure 5d), a flavonoid found in *Maclura pomifera* , *M. tinctoria* , and the leaves of *Psidium guajava* (Unuofin and Lebelo 2020), is an activator and sensitizer of the insulin receptor via stimulation of the glucose metabolic pathways. Morin improves diabetes by downregulating miR‐29a (Salehi et al. 2019), lowering blood glucose, regulating enzymes, and upsurging insulin (Unuofin and Lebelo 2020). It also reduced the surge of TNF‐α, IL‐1β, and IL‐6 via the sphingosine kinase 1 pathway. It reduced endothelial dysfunction by activating AKT/endothelial nitric oxide synthase signaling and attenuating ER stress through downregulating the PERK‐eIF2α‐ATF4 pathway by interacting with the PERK protein (Salehi et al. 2019). Morin also inhibited the proliferation of rat glomerular MCs and the aggregation of fibronectin induced by HG by suppressing the activation of the p38 MAPK and JNK signaling pathways (Hu et al. 2021).

#### 3.4.5 Fisetin

Fisetin (3,3′,4′,7‐Tetrahydroxyflavone) (Figure 5e) is a flavonol abundant in apples, grapes, cucumbers, and onions (Oladele et al. 2020). It reduces blood glucose and improves glucose homeostasis by inhibiting gluconeogenic enzymes and increasing glyoxalase 1 level/activity (Salehi et al. 2019). Its ability to mitigate diabetic complications, including its renoprotective efficacy, was reported (Dewanjee et al. 2020). In an experimental mouse model, fisetin reduced serum uric acid, creatinine, and blood urea nitrogen levels (Du et al. 2022).

### 3.5 Isoflavones

#### 3.5.1 Genistein

**FIGURE 6:**
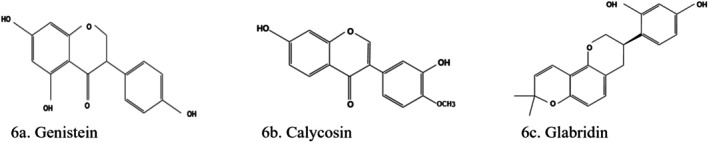
Structures of isoflavones: (a) Genistein, (b) calycosin, (c) glabridin. The structures were drawn using Kingdraw.

Genistein, also known as 4′,5,7‐Trihydroxyisoflavone (Figure 6a), is a naturally occurring isoflavonoid present in *Glycine max* (soybeans) (Unuofin and Lebelo 2020) and other leguminous plants (Hu et al. 2021). Genistein sustains the mass of the islet of Langerhans by increasing the number of β‐cells (Wickramasinghe et al. 2021). It protects β‐cells by initiating ERK1/2 and PKA, which result in increased insulin sensitivity and enhanced insulin action via the initiation of AMPK (Unuofin and Lebelo 2020). Genistein boosts tyrosine kinase inhibitors effects on DKD by upregulating renal phosphotyrosine expression and the ratio of renal phospho‐ERK/ERK. Genistein supplementation prevents DKD by inactivating the monocyte chemoattractant protein‐1 and NF‐kB pathways. It also downregulated fibrosis‐related marker expression, including protein kinase CβII, protein kinase C, and TGFβ‐1 (Hu et al. 2021).

Genistein improves renal fibrosis by regulating the TGF‐β/Smad3 pathway and reducing OS by activating the Nrf2‐HO‐1/NQO1 pathway (Putra et al. 2023). Genistein inhibits AGEs formation and exhibits α‐glucosidase inhibitory activity (Pan et al. 2022). It also improved kidney functions, reduced blood glucose and serum creatinine levels, increased mitochondrial membrane potential, downregulated the expression of MAPK, p53, p65, and NOX4, reduced OS, sustained podocyte integrity, and expanded the mesangial matrix (Kaabi 2022).

#### 3.5.2 Calycosin

Calycosin (3′,7‐dihydroxy‐4′‐methoxyisoflavone) (Figure 6b) is an isoflavone phytoestrogen extracted from *Astragalus membranaceus*. It has anti‐inflammatory, antioxidant, and antidiabetic properties. Calycosin improved renal function, attenuated tubular injury, inhibited renal fibrosis in diabetic mice in vivo, and increased the viability of HG‐induced HK‐2 cells in vitro via reduction of lactate dehydrogenase, MDA, and lipid ROS levels; increased GSH activity; promoted GPX4 expression; and inhibited nuclear receptor coactivator 4 expression in DKD models (Wei et al. 2023). Calycosin had significant ameliorative effects on diabetes‐induced renal inflammation by inhibiting NF‐kB‐dependent signaling in in vivo and in vitro experiments (Zhang et al. 2019). Another study showed that calycosin prevented the HG cytotoxicity induced in NRK‐52E by regulating the SIRT‐3/SOD2 pathway (Jiang et al. 2021). It also attenuated renal ischemia/reperfusion (IRI) injury in a male mice IRI model via suppression of NF‐κB‐mediated inflammation through PPARγ/early growth response 1 signaling (Zhang et al. 2022). A study involving STZ diabetes‐induced rats showed the ability of calycosin to hinder the development of DKD via the modulation of the NF‐κB/p65/NLRP3/TXNIP inflammasome cascade (Yosri et al. 2022). Calycosin rescued HG‐induced ferroptosis in HK‐2 cells by reducing lipid ROS and free iron import in the cells (Huang et al. 2022).

#### 3.5.3 Glabridin

Glabridin (Figure 6c) is a prenylated isoflavone extracted from the rhizomes and roots of *Glycyrrhiza glabra* L. (Oladele et al. 2020). It is the most effective antioxidant in liquorice extract (Kataya et al. 2011; Matsathit and Khimmaktong 2021). Glabridin has hepatoprotective, antidiabetic, neuroprotective, antioxidant, anti‐inflammatory, and antiproliferative effects (El‐Ghffar 2016; Hüseyin Aşkın Akpolat et al. 2023; Teoh and Das 2018). Several signaling mechanisms (MAPK, NF‐κB, Wnt/β‐catenin, PI3K/AKT, ERα/SRC‐1, and AMPK) are implicated in the regulatory functions of glabridin (Zhang, Wu, et al. 2023). It reverses hyperglycemia via its α‐glucosidase inhibitory effect, its ability to bind and activate PPARc (Teoh and Das 2018), and attenuation of diabetes‐induced damage to kidney function and structure via increased GSH, SOD, and CAT activities. Other activities include upregulated SLC7A11, GPX4, and SLC3A2 expression; downregulated VEGF, transferrin receptor 1, pAKT, and p‐ERK1/2 expression; and decreased iron and MDA concentrations in vitro and in vivo. Thus, this delay in DKD progression is associated with the inhibition of OS, ferroptosis, and the VEGF/AKT/ERK signaling pathway (Tan et al. 2022; Wei et al. 2023).

In a pilot study of people without diabetes and those with T2D, glabridin exhibited various positive metabolic effects and improved vascular functions, more in healthy volunteers than in those with T2D (Hattori et al. 2019). Glabridin mediates VEGF/AKT/ERKs–SLC7A11/SLC3A2 signal crosstalk, thus downregulating transferrin 1 expression of protein, decreasing neutrophil gelatinase‐associated lipocalin, and kidney injury molecule‐1 levels. Hence, it inhibits ferroptosis and ameliorates DKD renal injury (Chen et al. 2024).

### 3.6 Anthocyanidins

#### 3.6.1 Proanthocyanidins

**FIGURE 7:**
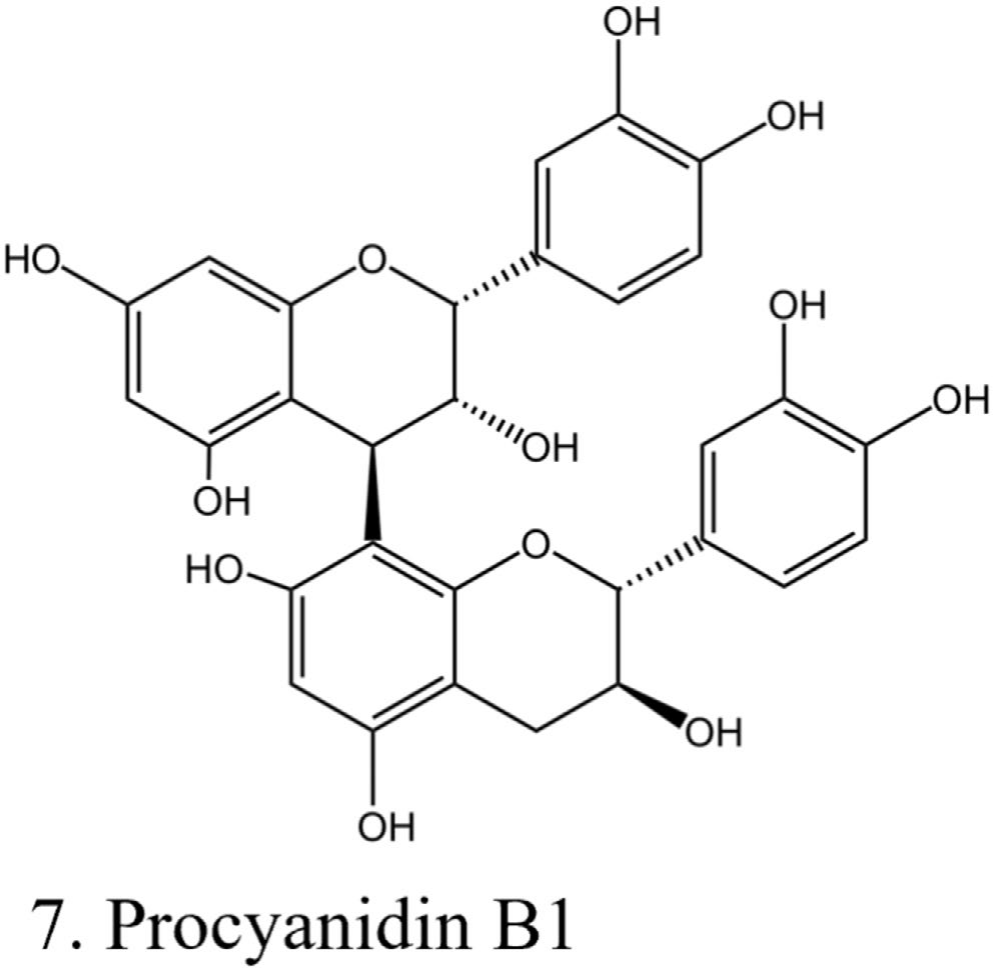
Structure of procyanidin B1. The structures were drawn using Kingdraw.

An example of proanthocyanidins (procyanidin B1) is shown in Figure 7. Proanthocyanidins are polyflavonoid tannins (condensed tannins) (Maugeri et al. 2022; Patane et al. 2023; Saad et al. 2022). There is an important class of bioflavonoids found in grape seed, French maritime pine bark, and many other plants. They have strong antioxidant activity (Hu et al. 2021; Li et al. 2019) and play an inhibitory role on lipid oxidation (Quan et al. 2022). Proanthocyanidins derived from grape seed extracts exerted inhibitory activity on podocyte injury in DKD in a rat model via activation of the AMPK‐silent information Regulator 1 (SIRT1)‐PGC1α pathway (Shrikanth and Nandini 2020). They exert anti‐inflammatory action by inhibiting the NF‐κB signaling cascade activation through MAP kinases, thus reducing the release of IL‐6, IL‐1β, and IL‐8 (Mohany et al. 2021).

The antidiabetic, renoprotective, cardioprotective, and antiatherosclerotic activities of proanthocyanidins were also documented (Mohany et al. 2021). Proanthocyanidins activate the Nrf2 signaling pathway and increase levels of SOD, total antioxidant capability, and GSH. Proanthocyanidins upregulate the expression of Nrf2, HO‐1, GSH *S*‐transferase, and NADH:ubiquinone oxidoreductase, which culminate in the reversal of renal damage in STZ‐induced DKD in rats. Proanthocyanidins also protect renal function and attenuate ER stress‐induced apoptosis through the Caspase‐12 pathway. They also decrease the expression of the AGE/RAGE axis and increase the expression of nephrin in diabetic rats (Hu et al. 2021). A study (Laddha and Kulkarni 2019) highlighted different types of proanthocyanidins from various plant sources, their effect on DKD, and the underlying mechanisms.

Anthocyanins are a glycosidic form of anthocyanidins present in *Myrciaria cauliflora* . They exert their effects on DKD through the inhibition of glomerular hypertrophy. Anthocyanins are also rich in *H. sabdariffa* , blueberries, raw cocoa beans, and other plants. They have antidiabetic properties via regulation of insulin secretion, improved insulin sensitivity, and glucose uptake in the adipose tissues, liver, and skeletal muscles. Therefore, they lower blood glucose through the AMPK, GPR40, glucose transporter 4 (GLUT4), and PI3K/AKT pathways (Salleh et al. 2021).

### 3.7 Chalcones

#### 3.7.1 Isoliquiritigenin

**FIGURE 8:**
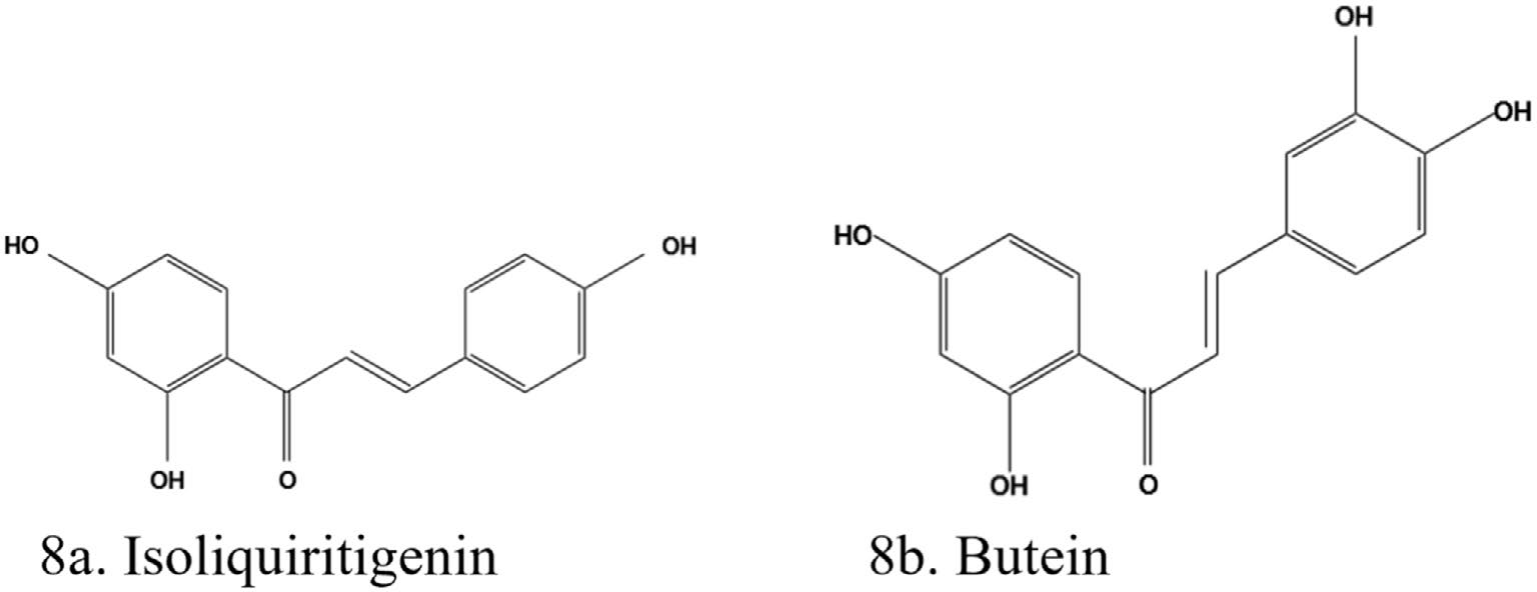
Structures of chalcones. (a) Isoliquiritigenin; (b) Butein. The structures were drawn using Kingdraw.

Isoliquiritigenin (2′,4,4′‐Trihydroxychalcone) (Figure 8a), a flavonoid extracted from *Glycyrrhiza* species, has multiple biological activities, which include antidiabetic, antioxidant, anti‐inflammatory, and antitumor effects. Isoliquiritigenin identified in liquorice reduced the accumulation of mesangial matrix in HG‐induced mesangial fibrosis by retarding TGF‐β1/Smad signal transduction (Li et al. 2010). Isoliquiritigenin reduced intercellular adhesion molecule‐1 and IL‐6 via inhibition of the JAK2/STAT3 pathway, relieving OS, renal fibrosis, and alleviating acute kidney injury in a diabetic rat model (Liu, Wang, Zhang, et al. 2023). Isoliquiritigenin suppresses NLRP3 inflammasome by reducing the release of high mobility group box 1, thus ameliorating fibrosis (Zhang et al. 2020). It also prevents hyperglycemia‐induced renal insults by the inhibition of OS and inflammation through a SIRT1‐dependent mechanism (Huang et al. 2020).

Other pathways documented for the renoprotective effect of isoliquiritigenin are control of NLRP3 expression, normalizing Sirt‐1/NF‐κB balance (Alzahrani et al. 2020), inhibition of epithelial–myofibroblast transdifferentiation, suppression of the TGFβ/STAT3 mechanism, which inhibited collagen secretion (Lin, Lin, et al. 2022; Lin, Ma, and Wang 2022), and activation of the Nrf2 mechanism (Al‐Qahtani et al. 2022).

#### 3.7.2 Butein

Butein (2′,3,4,4′‐Tetrahydroxychalcone) (Figure 8b), a natural phenolic chalcone isolated from *Toxicodendron vernicifluum* , *Semecarpus anacardium* , *Dalbergia odorifera*, *Cyclopia subternata*, and *Creopsis tungtoria* and other plants, inhibits central NF‐κB signaling and improves glucose homeostasis (Salehi et al. 2019). It also has anti‐inflammatory, antiangiogenic, antinephritic, antithrombin, and hepatoprotective activities as observed from different animal models (Semwal et al. 2015). Butein had an antidiabetic effect in the STZ‐induced animal model via repression of PPARγ (Prabhu and Rajeswari 2020). It exerted renoprotective effects in a mice model of cisplatin‐induced nephrotoxicity via alleviation of OS (Saraf and Saraf 2017). Butein was identified as one of the bioactive principles in *Coreopsis tinctoria* Nutt that had DKD protective effects based on an analysis of the network pharmacology of the plant active ingredients. Thirty‐four signaling pathways were identified to be closely related to DKD. Of these, the top pathways were PI3K/AKT, insulin, mTOR, and IR (Tian Li et al. 2022).

### 3.8 Flavonolignan

#### 3.8.1 Silymarin

**FIGURE 9:**
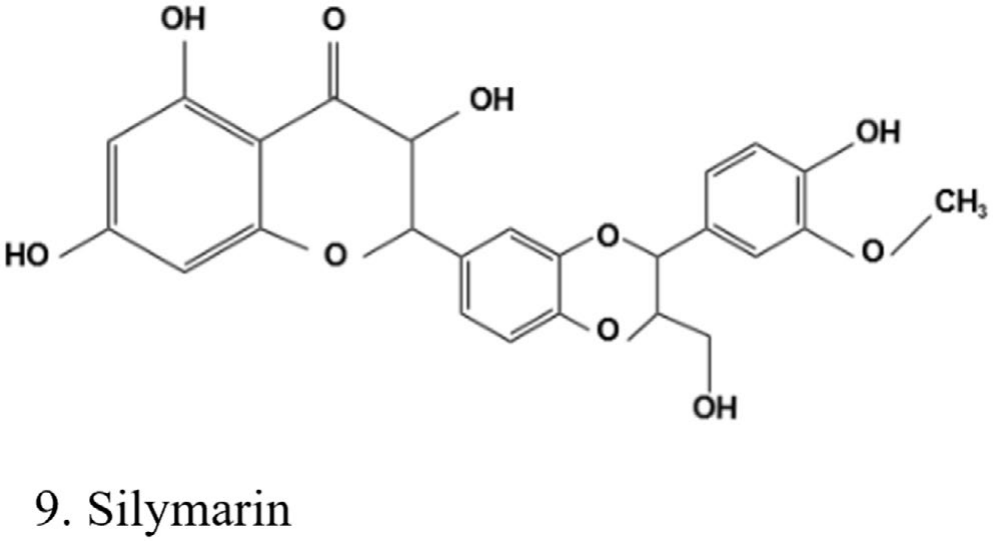
Structure of a flavonolignan, silymarin. The structures were drawn using Kingdraw.

Silymarin (Figure 9) isolated from the milk thistle plant ( *Silybum marianum* ) is a complex of flavonoids containing silydianin, silybin, and silychrisin, reduces blood glucose levels and shows renoprotective properties in T2D (Dewanjee et al. 2020; Salehi et al. 2019; Tang et al. 2021). It ameliorates diabetic cardiomyopathy by inhibiting TGF‐β1/Smad signaling (Salehi et al. 2019). Other properties include antioxidant, antifibrosis, anti‐inflammatory, and cell regeneration effects. Silymarin improved OS, renal fibrosis, podocyte injury, and inflammation in experimental diabetic rats via inhibition of TGF‐β/Smad and JAK2/STAT3/suppressor of cytokine signaling 1 pathways (Liu, Wang, Zhang, et al. 2023). Silymarin supported the growth of β‐cells and the production of insulin by activating Nkx6.1 and insulin mRNA (Dewanjee et al. 2020). Silymarin has preventive and therapeutic effects on diabetes and tubulointerstitial fibrosis. Chronic treatment with silymarin nanoliposomes effectively ameliorated inflammation, OS, and renal fibrosis via inhibiting JAK2/STAT3/suppressor of cytokine signaling 1 and TGF‐β/Smad signaling pathways in diabetic rats with STZ‐induced kidney injury (Chen et al. 2021).

## 4 Classification of Phenolic Acids and Derivatives and Their Molecular Mechanisms of Action

### 4.1 Cinnamic Acids and Derivatives

#### 4.1.1 Chlorogenic Acid

**FIGURE 10:**
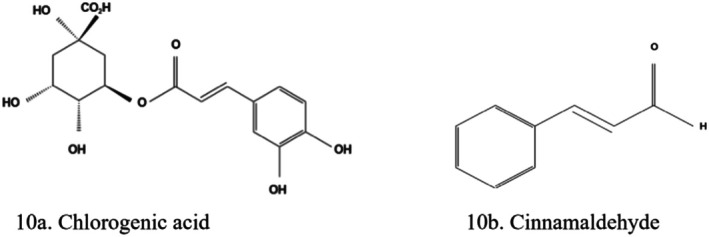
Structure of cinnamic acid derivatives. (a) Chlorogenic acid, (b) cinnamaldehyde. The structures were drawn using Kingdraw.

Chlorogenic acid (3‐*O*‐caffeoylquinic acid) (Figure 10a) stimulates glucose transport in skeletal muscles via AMPK activation (Naz et al. 2023; Salehi et al. 2019). It has antioxidant (Boonphang et al. 2021; Jin et al. 2023) and antidiabetic (Abu‐Odeh and Talib 2021; Andrade‐Cetto et al. 2019; Boonphang et al. 2021; Zaid et al. 2018; Zanzabil et al. 2023) Chlorogenic acid exerts its antihypertensive, antithrombotic, antiplatelet, and antioxidant activities by mediating the A2A receptor, NF‐κB, and the adenylate cyclase/cAMP/PKA mechanisms, thus improving cardiovascular diseases (Ali et al. 2020). Chlorogenic acid acts through different mechanisms on glucose metabolism. These include preventing glucose release by inhibiting the expression and activity of glucose‐6‐phosphatase, reducing glucose absorption, and enhancing insulin functionality by promoting AMPK phosphorylation.

Chlorogenic acids prevent and treat diabetic complications via a reduction of OS mechanism (Espinoza‐Hernandez and Andrade‐Cetto 2022). Chlorogenic acid identified in *H. sabdariffa* regulated blood pressure in patients with prerenal hypertension (Tienda‐Vazquez et al. 2022). It also lowered fasting blood glucose, serum creatinine, blood urea nitrogen, MDA, proteinuria, IL‐6, TNF‐α, IL‐1β, and NF‐κB; elevated GSH‐Px, CAT, Bcl‐2, chemokine receptors, SOD, Nrf2, and HO‐1 levels in different STZ‐induced DKD diabetic models. It promotes the formation of the antiapoptosis factor Bcl‐2 and attenuates the proliferation of MCs/mesangial expansion (Jin et al. 2023).

Mulberry leaf extract and neochlorogenic acid (a type of chlorogenic acid) attenuated glucolipotoxicity‐induced DKD in high‐fat diet‐fed diabetic mice (Hung et al. 2023).

Cryptochlorogenic acid, a type of chlorogenic acid and a major active ingredient in mulberry leaves, is effective at inhibiting ferroptosis via modulation of cystine/glutamate transporter (XC‐)/GSH peroxidase 4 (GPX4)/Nrf2 systems and inhibition of nuclear receptor coactivator 4 (NCOA4), thus decreasing islet insult in diabetes (Liu, Wang, and Gu 2023; Prasad et al. 2023).

#### 4.1.2 Cinnamaldehyde

Cinnamaldehyde is a cinnamic acid derivative (Figure 10b), a water‐soluble polyphenol compound, isolated from cinnamon ( *Cinnamomum verum* and *C. zeylanicum* ). The antidiabetic activity of cinnamaldehyde is attributed to its ability to reduce IR and promote hepatic glycogenesis (Unuofin and Lebelo 2020). In several clinical trials reported by Unuofin and Lebelo (Unuofin and Lebelo 2020), cinnamon showed a moderate reduction in fasting blood glucose, which was better than in the placebo groups. Cinnamaldehyde has antihyperglycemic, antihyperlipidemic, and anti‐obesity effects. Cinnamon oil is composed of more than 98% cinnamaldehyde and reduces hyaline casts, glomerular expansion, and tubular dilation in the kidney of alloxan‐induced renal damage in rats. This effect was observed in a dose‐dependent manner (Parveen et al. 2018). Cinnamaldehyde identified in *C. verum* J. has an antidiabetic effect (Skalli et al. 2019).

Cinnamtannin B1, another compound present in cinnamon, is a potent polypharmacological agent because of its ability to regulate three or more antidiabetic drug targets (Pereira et al. 2019).

### 4.2 Tannins

Tannins are astringent polyphenols found in many parts of plants (Maugeri et al. 2022; Amarasiri et al. 2020). The major sources of tannins are kola nuts (*Cola vera*), guarana ( *Paullinia cupana* ), tea ( *Camellia sinensis* ), coffee (*Coffea* spp.), and cocoa ( *Theobroma cacao* ) (Amarasiri et al. 2020; Sharma et al. 2019). Other rich sources of tannins are *Sericea lespedeza* and sorghum (*Sorghum bicolour*) (Sharma et al. 2019). Tannins are classified mainly as hydrolysable tannins and condensed tannins (non‐hydrolyzable) (Sharma et al. 2019). Tannins have various pharmacological activities, including antidiabetic (Wasana et al. 2021), wound healing, antibacterial, antiviral, antidiarrheal, antiparasitic, antihaemorrhoidal (Adnan et al. 2021), anti‐inflammatory, antitoxic, anticancerous, anthelmintic, antiallergic (Jing et al. 2022; Sharma et al. 2019), and antidiarrheal effects (Sahakyan et al. 2022).

Tannins contribute to lowering blood glucose (Kaabi 2022) by stimulating insulin secretion, reducing carbohydrate absorption by impeding α‐glucosidase and α‐amylase, enhancing β‐cell propagation and restoration, and preventing β‐cell impairment through free radical scavenging effects (Yikna and Yehualashet 2021). Tannins derived from pomegranate seeds significantly ameliorated the pathological changes in renal fibrosis induced by hyperglycemia in STZ‐induced diabetic rats. The underlying mechanism was attributed to the activation of microRNA‐495 through the enhancement of Smad7 (Li et al. 2017).

Tannic acid is a polymer of gallic acid that occurs naturally as a tannin polyphenol. It has antidiabetic and renoprotective effects (Chandak et al. 2009). The renoprotective effect of tannic acid results from the modulation of the NF‐κB/Nrf2 pathway, upregulation of Nrf2 and SOD2, an increase in Gpx1 expression, improvement in altered kidney biomarkers, inhibition of DNA strand breaks and fragmentation, and micronuclei induction (Basist et al. 2022). It also downregulates the renal expression of TGF‐β1, reduces ER stress, microRNA‐associated fibrosis, elevated glyoxalase 1 activity, and Nrf2 adjustment (Putra et al. 2023).

#### 4.2.1 Gallic Acid

**FIGURE 11:**
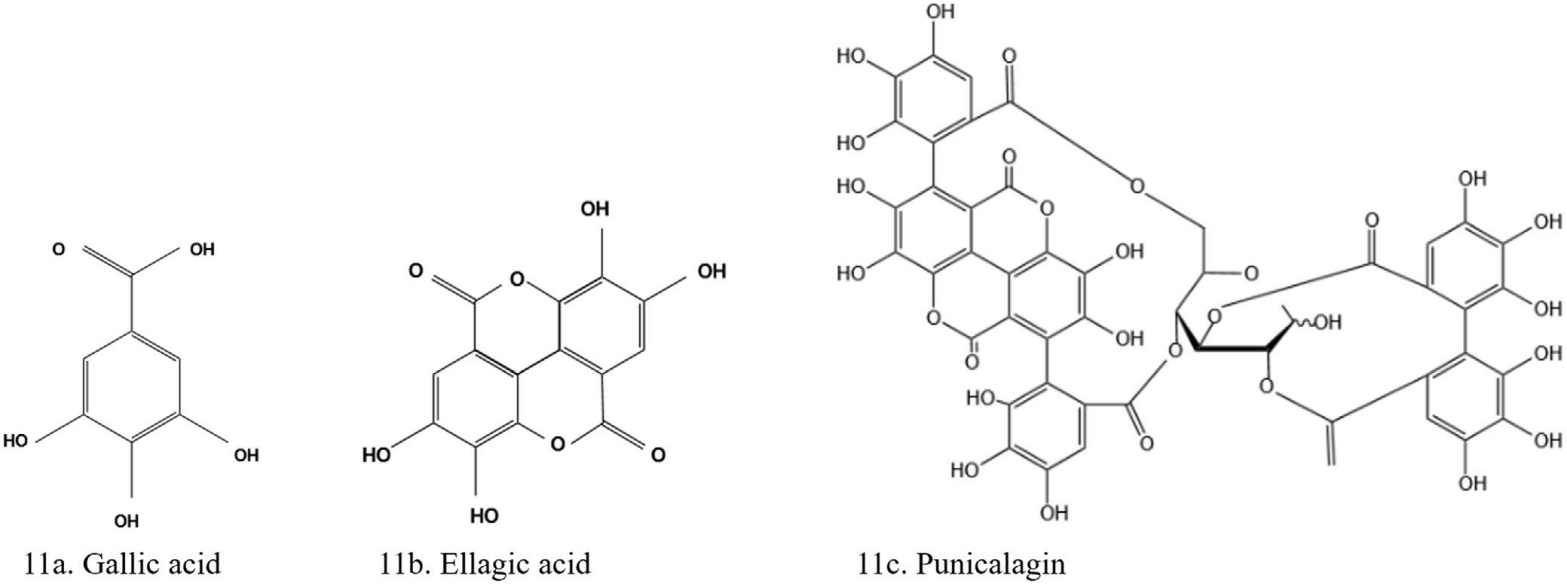
Structure of tannins. (a) Gallic acid, (b) ellagic acid, (c) punicalagin. The structures were drawn using Kingdraw.

Gallic acid (3, 4, 5 Trihydroxybenzoic acid) (Figure 11a), a building block of tannins, is found in grapes, green tea, red wine, oak bark, witch hazel, sumac, etc. Gallic acid has immense ameliorative effects on diabetes and its complications, including DKD, by acting on molecular pathways and vital targets involved in the disease progression (Maugeri et al. 2022). The mechanisms observed include increased OS, hexosamine pathway, polyol pathway, activation of PKC, TGF‐β, NF‐κβ, poly ADP‐ribose polymerase (PARP), IL‐1β, IL‐6, MAPK, and VEGF (Laddha and Kulkarni 2019; Sahakyan et al. 2022). Its strong antioxidant and anti‐inflammatory effect against renal damage results from its regulation of renal SOD, CAT, reduction of MDA, protein carbonyl, serum glutamate oxaloacetate transaminase, serum glutamate pyruvate transaminase, creatinine, uric acid, urea, and IL‐1β gene expression (Basist et al. 2022). Gallic acid prevents high gluco‐lipid‐induced β‐cell dysfunction by inhibiting apoptosis and restoring pancreatic and duodenal homeobox 1 and insulin expressions (Dewanjee et al. 2020).

#### 4.2.2 Ellagic Acid

Ellagic acid is a dilactone acid found in a wide range of plants (Salehi et al. 2019) (Figure 11b), such as raspberries (fruit of *Rubus idaeus* L.), blackberries ( *Rubus fruticosus* ), cloudberries ( *Rubus chamaemorus* L.), and strawberries; seeds, such as walnuts ( *Juglans regia* L.) and pecans ( *Carya illinoinensis* ); beverages, such as oak‐aged red wine and cognac obtained from grapes ( *Vitis vinifera* L.), pomegranates ( *Punica granatum* L.), peaches ( *Prunus persica* L.), persimmons ( *Diospyros kaki* L.), and plums ( *Prunus domestica* L.) (Rios et al. 2018). Ellagic acid possesses superior antioxidant properties (Lankatillake et al. 2019), genotoxicity prevention, antidiabetic activity, IR reduction (Salehi et al. 2019), antibacterial (Aryal et al. 2021), antilipidemic, and cardioprotective potentials (Wasana et al. 2021). It is also an effective inhibitor of ACE (Chakraborty and Roy 2021). Its antidiabetic effect is via action on β‐cells to stimulate insulin secretion, thus decreasing glucose intolerance.

The renoprotective effect of ellagic acid was reported in different experimental models, including human studies (Amor et al. 2020). In addition, Lin et al. (2021) observed the protective efficacy of ellagic acid on MCs from HG‐induced insult in a concentration‐based pattern by lowering the expression levels of the downstream transcription factor Forkhead Box O3a and inhibiting the activation of PI3K/AKT signaling. Ellagic acid protected against STZ‐induced DKD in rats by its antioxidant and antidiabetic effects via upregulation/transactivation of Nrf2 (ALTamimi et al. 2021). It also inhibited TGFβ1/Smad‐induced fibrosis of the kidney in the injured diabetic kidney (SaribaŞ et al. 2022).

The renoprotective effect of ellagic acid was reported in different experimental models, including human studies (172). In addition, Lin et al. (173) observed the protective efficacy of ellagic acid on MCs from HG‐induced insult in a concentration‐based pattern by lowering the expression levels of Forkhead Box O3a and inhibiting the activation of PI3K/AKT signaling. Ellagic acid protected against STZ‐induced DKD in rats by its antioxidant and antidiabetic effects via upregulation/transactivation of Nrf2 (174). It also inhibited TGFβ1/Smad‐induced fibrosis of the kidney in injured diabetic kidney (175).

#### 4.2.3 Punicalagin

Punicalagin, an ellagitannin, is the main component of pomegranates ( *Punica granatum* ) peel (Venusova et al. 2021) (Figure 11c). Its antioxidant, anti‐inflammatory, and renoprotective properties are documented (Aladaileh et al. 2021; Karwasra et al. 2016; Li et al. 2019). It is also protective against DKD and capable of countering IR induced by high‐fat diets (Du et al. 2022). Punicalagin further alleviates DKD by the downregulation of NOX4 expression and inhibition of the TXNIP/NLRP3 pathway‐mediated pyroptosis (An et al. 2020).

## 5 Other Polyphenols and Their Molecular Mechanisms of Action

### 5.1 Stilbenes

#### 5.1.1 Resveratrol

**FIGURE 12:**
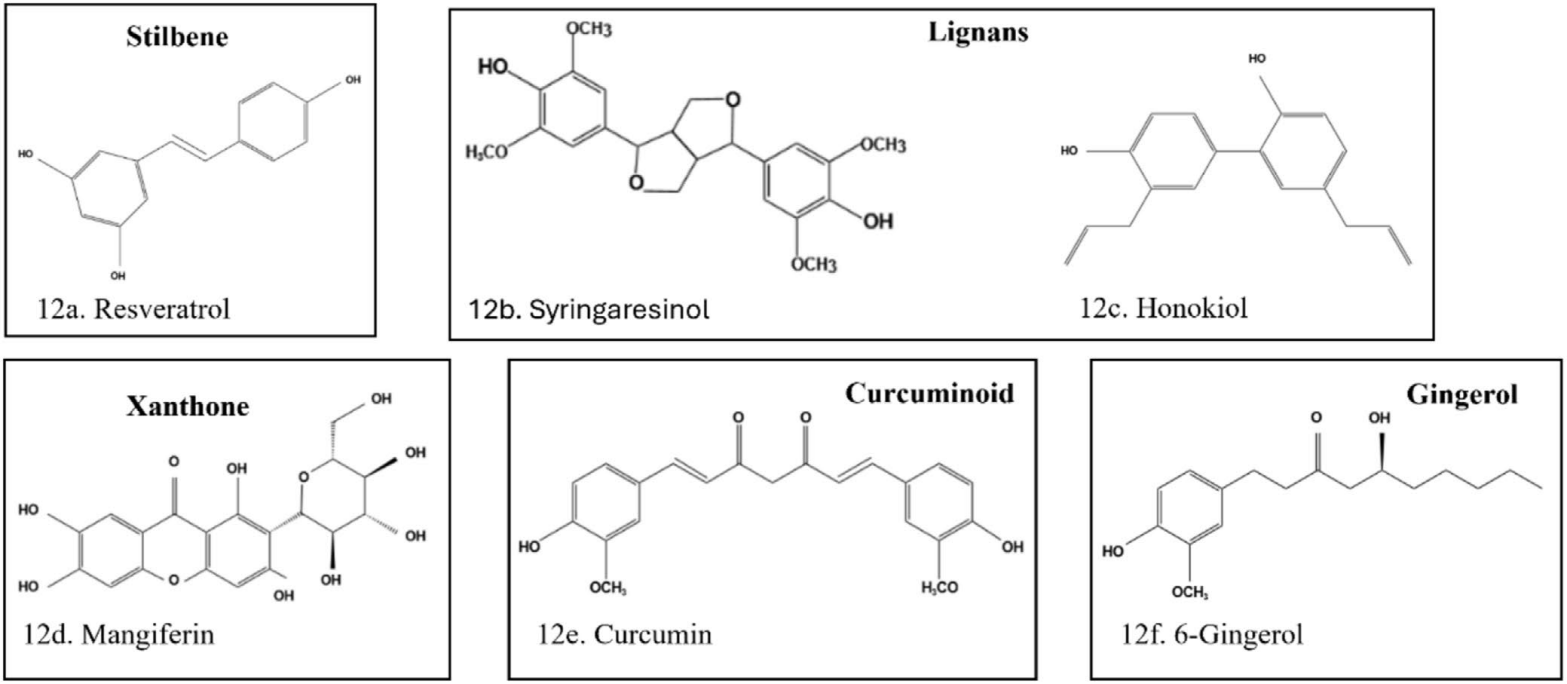
Structures of other polyphenols. (a) Resveratrol, (b) syringaresinol, (c) honokiol, (d) mangiferin, (e) curcumin, (f) 6‐gingerol. The structures were drawn using Kingdraw.

Resveratrol (3,4′,5‐Trihydroxystilbene) (Figure 12a) is a polyphenol and major bioactive component found in *Vitis vinifera* (grapevine) (Unuofin and Lebelo 2020) and blueberries (Yang et al. 2020). The compound reduces renal dysfunction (Du et al. 2022; Putra et al. 2023) and OS (Chen et al. 2022) and exerts anti‐inflammatory effects (Jin et al. 2023). Resveratrol upregulates the expression and activation of AMPK, which may have beneficial effects in the early stages of DKD (Dehdashtian et al. 2020; Shrikanth and Nandini 2020). Resveratrol suppresses diabetes‐induced inflammation and the proliferation of renal MCs through AKT/NF‐κB pathways (Avila‐Carrasco et al. 2021; Parveen et al. 2018). It also prevents experimental DKD by regulating PI3K/AKT components in kidney tissues (Rayego‐Mateos et al. 2020) and averting apoptosis of β‐cells influenced by islet amyloid polypeptide on culture medium (Unuofin and Lebelo 2020). Resveratrol ameliorates DKD‐linked sensorimotor disruptions by suppressing the activities of nicotinamide adenine dinucleotide phosphate (NADP) oxidase (Kaabi 2022). In a randomized, double‐blind, placebo‐controlled trial consisting of 60 patients with DKD, resveratrol (500 mg/day) and losartan (12.5 mg/day) compared well to placebo (500 mg/day) plus losartan (12.5 mg/day) and had more protective effects after a 90‐day intervention in patients. The study showed improvements in several kidney profile parameters (Tang et al. 2021). A similar result was observed for safflower yellow, earlier known as carthamine extracted from Flos Carthami ( *Carthamus tinctorius* L.) (Tang et al. 2021).

### 5.2 Lignans

#### 5.2.1 Syringaresinol

Syringaresinol is a polyphenolic lignan in plants like flax seed, *Brassica* vegetables, sesame seed, and grains (Figure 12b). It ameliorated the progression of DKD via the activation of Nrf2 and inactivation of TGF‐β1/Smad pathways. In diabetic C57BL/6 mice, syringaresinol also inhibited pyroptosis by activating the Nrf2 antioxidant pathway (Putra et al. 2023).

#### 5.2.2 Honokiol

Honokiol (5,3′‐Diallyl‐2,4′‐Dihydroxybiphenyl) (Figure 12c), another polyphenol lignan with antidiabetic activity, is identified in magnolia plant species (*Magnolia officinalis*). It increases phosphorylation and factors associated with downstream insulin signaling (Chaudhary 2020). Honokiol protects pancreatic β‐cells against HG and intermittent hypoxia‐induced injury by activating the Nrf2/ARE pathway in vitro and in vivo (Salehi et al. 2019). Honokiol is a SIRT3 activator shown to lower the level of ECM components and alpha‐smooth muscle actin in renal fibrosis induced by unilateral ureteral obstruction in experimental mice. Suppression of SIRT3 is associated with the emergence of EMT. Honokiol thus prevents fibrosis through the inhibition of the EMT process. Honokiol also promotes NF‐κB and p65 phosphorylation, which indicates that it may exert anti‐EMT activity by anti‐inflammatory mechanisms. Reduced Smad2 and 3 phosphorylation was also observed in kidneys treated with honokiol (Liu, Sun, et al. 2022). A derivative of honokiol called 4‐*O*‐methylhonokiol had a kidney protective effect in DKD by activating AMPK/PGC‐1α/carnitine palmitoyltransferase 1B and Nrf2/SOD2 pathways (Chen et al. 2022).

### 5.3 Xanthones

#### 5.3.1 Mangiferin

Mangiferin, a polyphenolic bioactive compound, is mostly found in *Mangifera indica* (mango) (Figure 12d). Mangiferin has antidiabetic, hypolipidemic, and antioxidant properties (Wei et al. 2023). It inhibits the anaerobic metabolism of pyruvate to lactate and enhances pyruvate oxidation. Mangiferin also prevents the progression of DKD and improves renal function in a rat model of the disease and cultured rat MCs (Adinortey et al. 2019; Putra et al. 2023). It also exhibits its renoprotective effect (Jin et al. 2023; Wei et al. 2023) by inhibiting oxidative tension. Mangiferin protected podocytes in diabetic rats by upregulating autophagy through the AMPK‐mTOR‐ULK1 cascade.

### 5.4 Curcuminoids

#### 5.4.1 Curcumin

Curcumin is a polyphenolic compound (Figure 12e) obtained from *Curcuma longa* (turmeric). It exhibits high antidiabetic (Kaabi 2022; Omale et al. 2023; Unuofin and Lebelo 2020), antioxidant (Chen et al. 2023), anti‐inflammatory (Chen et al. 2022), antiobesity (Ansari et al. 2022; Basu et al. 2023; Saad et al. 2022), antitumor (Kaabi 2022), and antiaging properties (Lin et al. 2024). Of these properties, its anti‐inflammatory action is considered the basis for its therapeutic effects in different diseases (Kaabi 2022). Curcumin reduces plasma glucose and glycosylated hemoglobin levels by regulating the polyol pathway. It activates liver enzymes involved in gluconeogenesis, glycolysis, and lipid metabolism (Cox‐Georgian et al. 2019) and has renoprotective properties (Dehdashtian et al. 2020; Du et al. 2022; Tienda‐Vazquez et al. 2022). A study (Naz et al. 2023) reported the antidiabetic activity of curcumin in various experimental models of diabetes and their underlying mechanisms. Curcumin suppressed EMT through the regulation of Smad and non‐Smad pathways, attenuated liver and renal EMT via inhibition of the PI3K/AKT/mTOR pathway, inhibited TGF‐β‐activated kinase 1, and downregulated the expression of JNK and p38 in peritoneal EMT. It also regulated the tetratricopeptide repeat domain 3/Smad ubiquitination regulatory factor 2/Smad axis (Liu, Sun, et al. 2022).

The underlying mechanism for its anti‐inflammatory, antiapoptotic, and antidiabetic activities involves lowering TNF‐α, caspase‐3, IL‐1β, IL‐6, and Bax levels in vivo by blocking the phosphorylation of JNK and NF‐κB protein signaling pathways (Jin et al. 2023; Yang et al. 2020). Curcumin improves renal function and reduces proinflammatory cytokine production in DKD (Avila‐Carrasco et al. 2021; Rayego‐Mateos et al. 2020). Curcumin has been shown to lower TGF‐β levels in the urine and blood of people with DKD in clinical trials (Jaafarinia et al. 2022). Curcumin prevented OS and accumulation of ECM in DKD by ameliorating HG‐induced superoxide and the Wnt/β‐catenin pathway (Jiang et al. 2022). In a rat model of DKD, curcumin suppressed the expressions of renal inflammatory genes and lowered the phosphorylation of caveolin‐1 at Tyr14 and TLR4 expression. It also lowered HG‐induced caveolin‐1 phosphorylation, synthesis of TLR4, and proinflammatory cytokines (Kaabi 2022).

In a clinical study (Khan et al. 2022), curcumin at 600–1500 mg reduced OS in patients who have nondiabetic and diabetic proteinuric chronic kidney disease. The study also showed that treating patients with chronic kidney disease using *C. longa* with *Boswellia serrata* (824 and 516 mg, respectively) reduced prostaglandin E2 (PGE2) and ameliorated inflammation. In a different study, curcumin turned on Nrf2 and turned off NADPH oxidase, NF‐κB, the PKCβII/p66Shc signal, and NLRP3 inflammasome activity in people with DKD (Chen et al. 2022). The same study documented a significant ameliorative effect of curcumin in STZ‐induced DKD rats by regulating albumin/protein urea and increasing creatinine clearance, which involved activation of Nrf2, inhibition of NF‐κB, NADPH oxidase, and significant upregulation of the PKCβII/p66Shc signal. The study also found that curcumin can slow down the progression of DKD by stopping the activity of the NLRP3 inflammasome and lowering levels of neutrophil gelatinase‐associated lipocalin and kidney injury molecule 1.

Another clinical study that involved supplementing 2.5 g of curcumin for 3 days per week for 12 weeks in patients undergoing hemodialysis showed decreased mRNA expression of NF‐κB and protein C high‐sensitivity reactive (Reis et al. 2024). The study also reported that curcuminoid supplementation for 12 weeks suppressed lipid peroxidation and lowered uremic toxins in patients with DKD undergoing peritoneal dialysis. Different dosages of curcumin had a renoprotective effect in various experimental animal models of diabetes by targeting multiple mechanisms (Jin et al. 2023).

### 5.5 Gingerols

#### 5.5.1 6‐Gingerol

6‐gingerol is a polyphenol found in *Zingiber officinale* (ginger) (Figure 12f). It is hypolipidemic and anti‐inflammatory among many other functions (Oladele et al. 2020). 6‐gingerol can be converted into shogaols and zingerone. These active compounds, alongside paradol, are responsible for the therapeutic effects of ginger (Veisi et al. 2022). The hypoglycemic effects of ginger and its related components are attributable to its serotonin receptor‐blocking activity (Parveen et al. 2018). Gingerol increases glucose uptake by promoting the translocation of GLUT‐4 via AMPK activation in L6 myocytes. It also protects pancreatic β‐cells from OS, increases insulin receptor sensitivity, and enhances β‐cell function, which decreases IR. Gingerol was shown to regulate in vivo hepatic gene expression of enzymes involved in glucose metabolism, which resulted in a decrease in glucose production and an increase in glycogen synthesis, thus contributing to its antihyperglycemic effect (Adinortey et al. 2019; Almatroodi et al. 2021).

## 6 Conclusions

Preclinical and clinical studies show that phytomedicine is a promising source of therapy for DKD due to its diverse bioactive principles, including polyphenols. This review shows evidence from in vitro, in vivo, and clinical trials for the action of polyphenolic compounds on the intricate signaling mechanisms of DKD holistically. This comprehensive therapeutic protocol aligns with the complex network of pathways involved in the pathogenesis of the disease. The common mode of action of polyphenols on DKD reported in this review includes nephroprotective, antidiabetic, anti‐inflammatory, antifibrosis, hepatoprotective, antiobesity, antiangiogenetic, and antihypertension.

Although the different classes of polyphenols have DKD therapeutic effects, flavonoids have shown vast therapeutic potential. Nevertheless, among all polyphenols, curcumin, resveratrol, EGCG, and proanthocyanidins have been extensively explored in clinical trials for their protective effect on kidney disease.

The intricate signaling cascades of DKD are therapeutic intervention points for the disease, indicating the need for compounds that will match the mechanistic complexity. Polyphenols have vast biological properties and can target these mechanisms simultaneously and synergistically. Holistically, integrating polyphenols into conventional therapy could enhance targeting the underlying multi‐mechanisms of DKD. This would be a more resilient and comprehensive defense than classical medication alone—one that aligns more efficiently with the complexity of DKD and the unmet patient needs.

The future research directions should be focused on standardizing polyphenol formulations and elucidating synergistic interactions. There should also be clinical trials targeted at more polyphenols with nephroprotective effects from different experimental models, either alone or alongside established therapies. The long‐term effects at different dosages should be prioritized in future research. There is also a need to assess the variability in patient response and across different age ranges. These would facilitate the bench‐to‐bed application of polyphenols as a reliable therapeutic protocol for managing DKD and other metabolic disorders.

## Author Contributions

**Esienanwan E. Efiong:** conceptualization (lead), data curation (lead), investigation (lead), methodology (equal), writing – original draft (lead), writing – review and editing (equal). **Emmanuel Effa:** methodology (equal), resources (equal), validation (equal), writing – review and editing (equal). **Esther Peters:** methodology (supporting), software (equal), writing – review and editing (supporting). **Ochuko L. Erukainure:** visualization (equal), writing – review and editing (equal). **Peter U. Amadi:** methodology (equal), resources (equal), writing – review and editing (equal). **Joshua Onyeka Ikebuiro:** resources (supporting), writing – review and editing (equal). **Sapna Sharma:** methodology (equal), resources (equal), writing – review and editing (supporting). **Christoph Schmaderer:** methodology (equal), resources (supporting), validation (equal), writing – review and editing (equal). **Kathrin Maedler:** writing – review and editing (equal). **Emmy Tuenter:** formal analysis (equal), investigation (supporting), methodology (equal), resources (equal), supervision (equal), validation (equal), writing – review and editing (equal). **Harald Grallert:** project administration (lead), resources (lead), supervision (lead), writing – review and editing (supporting).

## Conflicts of Interest

The authors declare no conflicts of interest.

## Data Availability

The data that support the findings of this study are available in the Supporting Information of this article.
